# Efficacy and safety of dietary polyphenols in rheumatoid arthritis: A systematic review and meta-analysis of 47 randomized controlled trials

**DOI:** 10.3389/fimmu.2023.1024120

**Published:** 2023-03-22

**Authors:** Zhiyong Long, Wang Xiang, Qi He, Wei Xiao, Huagen Wei, Hao Li, Hua Guo, Yuling Chen, Mengxia Yuan, Xiao Yuan, Liuting Zeng, Kailin Yang, Yuxuan Deng, Zhen Huang

**Affiliations:** ^1^ Department of Rehabilitation Medicine, Guangzhou Panyu Central Hospital, Guangzhou, China; ^2^ The First People's Hospital of Changde City, Changde, China; ^3^ People's Hospital of Ningxiang City, Ningxiang, China; ^4^ Dental Materials Science, Applied Oral Sciences and Community Dental Care, Faculty of Dentistry, The University of Hong Kong, Hong Kong, Hong Kong SAR, China; ^5^ Guangzhou University of Chinese Medicine, Guangzhou, China; ^6^ Joint Shantou International Eye Center of Shantou University and The Chinese University of Hong Kong, Shantou University Medical College, Shantou, China; ^7^ Hunan University of Chinese Medicine, Changsha, China; ^8^ Department of Rheumatology and Clinical Immunology, Peking Union Medical College Hospital, Chinese Academy of Medical Sciences & Peking Union Medical College, Beijing, China; ^9^ Qiqihar Medical University, Qiqihar, China

**Keywords:** dietary polyphenols, rheumatoid arthritis, randomized controlled trial, systematic review, meta-analysis

## Abstract

**Objective:**

To evaluate safety and efficacy of dietary polyphenols in the treatment of rheumatoid arthritis (RA).

**Methods:**

CNKI, Pubmed, Cochrane library, Embase were searched to collect randomized controlled trials (RCTs) of dietary polyphenols in the treatment of RA. The databases were searched from the time of their establishment to November 8nd, 2022. After 2 reviewers independently screened the literature, extracted data, and assessed the risk of bias of the included studies, Meta-analysis was performed using RevMan5.4 software.

**Results:**

A total of 49 records (47 RCTs) were finally included, involving 3852 participants and 15 types of dietary polyphenols (Cinnamon extract, Cranberry extract, Crocus sativus L. extract, Curcumin, Garlic extract, Ginger extract, Hesperidin, Olive oil, Pomegranate extract, Puerarin, Quercetin, Resveratrol, Sesamin, Tea polyphenols, Total glucosides of paeony). Pomegranate extract, Resveratrol, Garlic extract, Puerarin, Hesperidin, Ginger extract, Cinnamon extract, Sesamin only involve in 1 RCT. Cranberry extract, Crocus sativus L. extract, Olive oil, Quercetin, Tea polyphenols involve in 2 RCTs. Total glucosides of paeony and Curcumin involve in more than 3 RCTs. These RCTs showed that these dietary polyphenols could improve disease activity score for 28 joints (DAS28), inflammation levels or oxidative stress levels in RA. The addition of dietary polyphenols did not increase adverse events.

**Conclusion:**

Dietary polyphenols may improve DAS28, reduce C-reactive protein (CRP) and erythrocyte sedimentation rate (ESR), and improve oxidative stress, etc. However, more RCTs are needed to verify or modify the efficacy and safety of dietary polyphenols.

**Systematic review registration:**

https://www.crd.york.ac.uk/prospero/, identifier CRD42022315645.

## 1 Introduction

Rheumatoid arthritis (RA) is a chronic, systemic, highly disabling autoimmune disease with non-infectious, symmetrical, progressive polyarthritis as the main clinical manifestation. It primarily affects the joints, but should be considered as a syndrome that includes extra-articular manifestations, such as rheumatoid nodules, pulmonary involvement, or vasculitis, as well as systemic comorbidities (1, 2). It is the most common systemic irritant arthritis and is one of the world’s major public health challenges, affecting approximately 1% of the world’s population (3). RA can occur at any age, and its incidence begins to increase significantly at age 25; at age 55, the incidence of rheumatoid arthritis peaks (4, 5). The prevalence of RA varies among different ethnic groups. The incidence of RA is 7 percent among Native Americans and 0.2 to 0.4 percent in some other countries (6). For example, the prevalence of RA in China is 0.2% to 4% (7), the number of patients is as high as 4 million, and the remission rate is roughly only 8.6% (8). Like most other autoimmune diseases, RA is more common in women than in men in a ratio of 2-3:1 (9).

Although RA has a high disability rate and high prevalence, its pathogenesis is not fully understood. At present, the treatment of RA has entered into a comprehensive management strategy, which aims to slow down the progression of the disease, reduce the occurrence of pain and bone destruction, preserve the joint mobility of patients as much as possible, and avoid disability (10, 11). The main treatments for RA include conventional synthetic antirheumatic drugs (DMARDs), glucocorticoids, non-steroidal anti-inflammatory drugs (NSAIDs), targeted synthetic DMARDs, bio-original DMARDs and biosimilar DMARDs (12, 13). Although the above drugs can significantly relieve clinical symptoms, they have a single target, many adverse reactions (such as allergic reactions and blood diseases), poor long-term efficacy, and high cost (6). Meanwhile, in the vast developing countries, due to the huge economic burden, it is currently difficult for most RA patients to receive standardized and effective treatment, which is accompanied by serious physical and mental injuries (11). As an important complementary alternative therapy, dietary supplements are now also an important adjunct option for RA patients. For example, dietary polyphenols have the characteristics of multi-target, multi-component, and multi-mechanism in pharmacology, and have definite clinical efficacy, as well as the advantages of less toxic side effects and no drug resistance (14).

Polyphenols are a diverse class of plant-derived compounds with water-soluble chemical properties (15). They are widely found in herbs, fruits, teas, red berries, coffee, red wine, and dark chocolate worldwide, and are well-known antioxidants and have been proposed as treatments for several inflammatory and metabolic disorders (16–19). Studies have shown that polyphenols can prevent oxidative stress, inhibit inflammation and modulate immunity and other pharmacological effects (20), especially inhibiting inflammation and immunity. These compounds may improve fibroblast-like synoviocytes (FLS) in rats with adjuvant arthritis (AIA) by inducing inhibition of several pro-inflammatory immunochemokines and promoting apoptosis through mitochondrial signaling pathways and endoplasmic reticulum stress (21). Currently, many randomized controlled trials (RCTs) of dietary polyphenols in the treatment of RA have been published. However, the results and treatment measures of these RCTs are different, which cannot provide a basis for clinicians to formulate treatment plans for RA. Therefore, a comprehensive and in-depth summary of these RCTs is urgently needed to complement the therapeutic modalities for the treatment of RA. Hence, this study was the first comprehensive systematic review and meta-analysis of RCTs on the treatment of RA with dietary polyphenols in order to provide high-quality evidence for clinicians.

## 2 Materials and methods

### 2.1 Protocol

This systematic review and meta-analysis were conducted strictly in accordance with the protocol registered in PROSPERO (CRD42022315645) and PRISMA-guidelines (see **Supplementary Materials**) (22).

### 2.2 Literature search strategy

The databases were searched from the time of their establishment to November 8nd, 2022. The databases include China National Knowledge Infrastructure (CNKI), Web of Science, Sinomed, VIP Database for Chinese Technical Periodicals, Medline Complete, ArXiv, Pubmed, Embase, Wanfang Database on Academic Institutions in China, ClinicalTrials.gov and Cochrane Library were searched from. The search strategy was shown in **Table S1**.

### 2.3 Inclusion and exclusion criteria

#### 2.3.1 Participants

Participants were RA patients. The diagnosis of RA conformed to the RA diagnostic criteria in the 2010 Rheumatoid Arthritis Diagnosis and Treatment Guidelines of the Rheumatology Branch of the Chinese Medical Association or the RA diagnostic criteria proposed by the American College of Rheumatology/2017 European Federation of Rheumatology in 1987 or other recognized criteria.

#### 2.3.2 Intervention

The experimental group was treated with dietary polyphenols, and the route of administration, preparation type, etc. were not limited; the treatment could be combined with conventional treatment or control group treatment. Treatment in the control group was conventional therapy or a placebo or other non-dietary polyphenol therapy (RCT researchers claimed to limit the consumption of polyphenol-rich diets in the control group.).

#### 2.3.3 Outcomes

(1) Efficacy indicators: Disease activity score for 28 joints (DAS28), American College of Rheumatology 20% (ACR20), American College of Rheumatology 50% (ACR50), American College of Rheumatology 70% (ACR70). (2) Inflammatory parameters in serum: C-reactive protein (CRP), erythrocyte sedimentation rate (ESR), tumor necrosis factor (TNF-α), interleukin (IL-6), rheumatoid factor (RF). (3) Oxidative stress markers: malondialdehyde (MDA) and total antioxidant capacity (TAC). (4) Adverse events.

#### 2.3.4 Study design

The study design included RCTs with no restrictions on publication time, language, quality and publication status.

#### 2.3.5 Exclusion criteria

(1) non-RCT; (2) review; (3) cohort study; (4) control group also used dietary polyphenol-enriched therapy

### 2.4 Literature quality evaluation and data extraction

Two reviewers independently searched the database according to the search strategy, and initially excluded studies that did not belong to the treatment of RA with polyphenols after reading the titles and abstracts. They then independently screened literature, assessed quality, and extracted data according to inclusion and exclusion criteria, and any disagreements were resolved by brainstorming with other reviewers. The data in the literature were registered and managed in Excel form, and the extracted data included the name of the investigator, the year of publication of the literature, the general characteristics of the included patients and the number of cases, intervention measures, outcomes, etc (23). The methodological quality of the included studies was assessed according to the Cochrane Collaboration’s Risk of Bias Assessment Tool (24). The main contents of the evaluation are: (1) the application of random allocation method; (2) the implementation of random concealment; (3) the implementation of blinding of research subjects and treatment plans; (4) the implementation of blinding of outcome measurers; (5) Integrity of Outcomes; (6) Selective reporting; (7) Other bias.

### 2.5 Statistical analysis

Meta-analysis was performed using Review Manager 5.4 provided by the Cochrane Collaboration. Enumeration data were expressed as risk ratio (RR), measurement data were expressed as weighted mean difference (WMD) or standard mean difference (SMD), and each effect size was expressed as 95% confidence interval (CI). The chi-square test was used to analyze the heterogeneity between the results. P>0.1 and I²≤50% was considered low heterogeneity; P<0.1 and I²>50% was considered high heterogeneity (25). Regardless of heterogeneity, due to the different sources of polyphenols, we use the random effects model for all outcome. Outcomes with included RCTs ≥ 4 were selected for subgroup analysis, sensitivity analysis, publication bias assessment, meta-regression analysis and GRADE score. Sensitivity analysis, publication bias assessment, meta-regression were analyzed by STATA 15.0; GRADE score was assessed by the GRADEprofiler software (26).

## 3 Results

### 3.1 Literature search results

**Figure 1:**
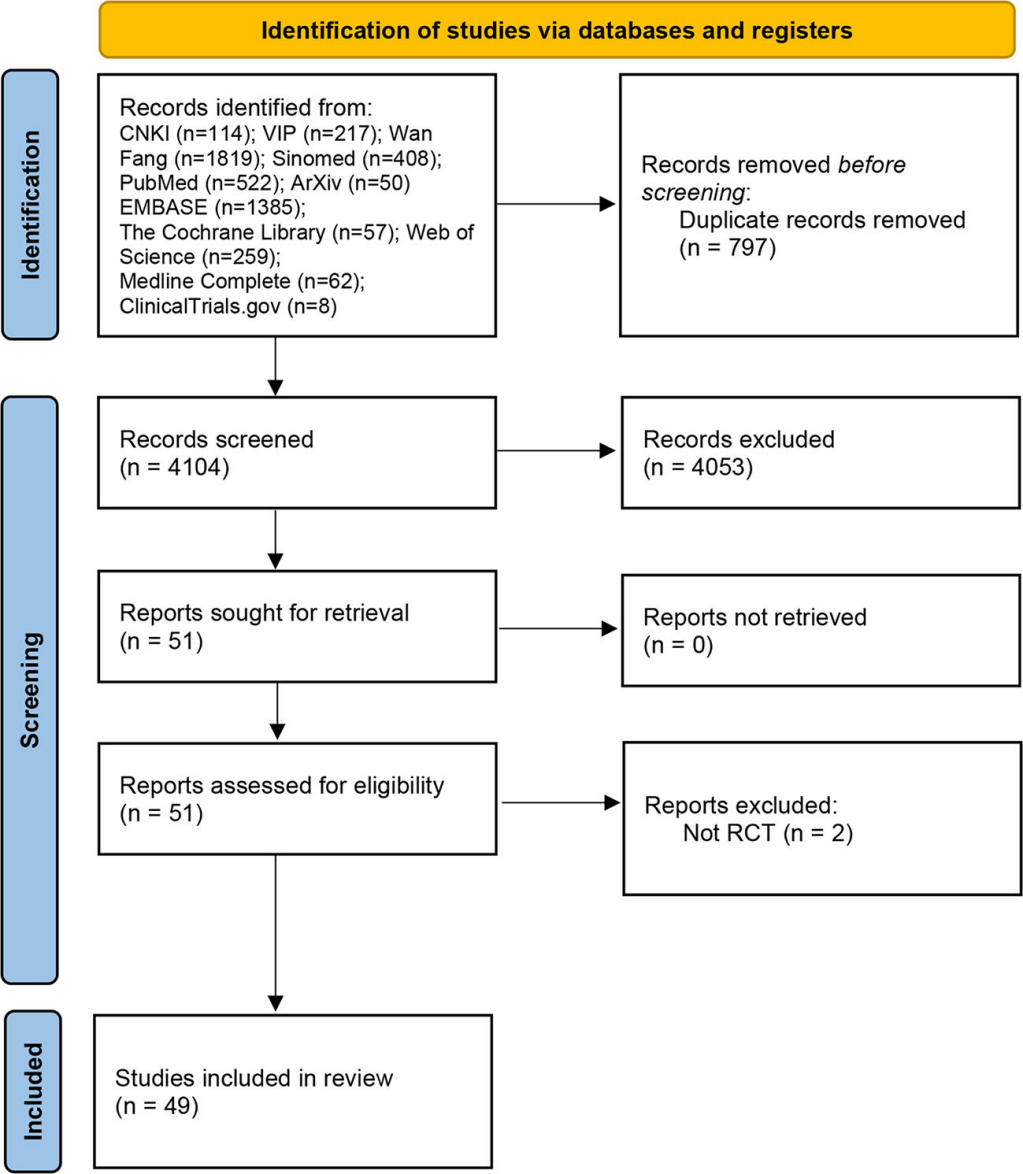
Flow diagram of clinical research.

A total of 53 relevant studies were obtained in the initial examination, and after screening, 49 records were finally included (27–76), and 2 records were excluded for they are not RCTs (77, 78). The literature screening process and results are shown in (**Figure 1**).

### 3.2 Description of included trials

Two records (28, 29) came from the same RCT and were therefore recorded as Javadi et al., 2017 (28, 29). Two records (32, 33) came from the same RCT and were therefore recorded as Moosavian et al., 2020 (32, 33). Some RCTs contain multiple groups and are therefore split into groups a and b. The included RCTs involved 15 dietary polyphenols (Cinnamon extract, Cranberry extract, Crocus sativus L. extract, Curcumin, Garlic extract, Ginger extract, Hesperidin, Olive oil, Pomegranate extract, Puerarin, Quercetin, Resveratrol, Sesamin, Tea polyphenols, Total glucosides of paeony) and were from 7 different countries (Iran, Korea, Egypt, China, Japan, Brazil, India). The details of study characteristics are presented in **Table 1**.

**Table 1:** The characteristics of the included studies.

Disease	Study	Country	Sample size		Intervention		Mean age (years)		Baseline DAS28		Baseline CRP (mg/L)		Baseline ESR (mm/h)		Relevant outcomes	Duration
			Trial group	Control group	Trial group	Control group	Trial group	Control group	Trial group	Control group	Trial group	Control group	Trial group	Control group		
Pomegranate extract	Ghavipour et al., 2016 (27)	Iran	30	25	Pomegranate extract (contained 40% ellagic acid) with no changes to current medication (mainly Methotrexate, Hydroxychloroquine, Sulfasalazine and Prednisolone)	Placebo with no changes to current medication (mainly Methotrexate, Hydroxychloroquine, Sulfasalazine and Prednisolone)	48.4 ± 11.4	49.1 ± 12.2	4.9 ± 0.8	4.7 ± 1.1	29.0 ± 15.6	30.6 ± 19.6	8.0 ± 4.2	6.6 ± 4.5	DAS28, ESR, CRP, Oxidative stress markers	8 weeks
Quercetin	Javadi et al., 2017 (28, 29)	Iran	20	20	Quercetin 500mg + conventional treatment (mainly Methotroxate, Hydroxychloroquine, Sulfasalazine, Cyclosporine, Prednisolone, NSAIDs)	Placebo + conventional treatment (mainly Methotroxate, Hydroxychloroquine, Sulfasalazine, Cyclosporine, Prednisolone, NSAIDs)	46.55 ± 9.94	48.00 ± 8.39	3.22 ± 0.93	3.13 ± 1.1	2.89 ± 2.95#	3.28 ± 2.32#	19.00 ± 8.62	21.10 ± 12.38	DAS28, ESR, CRP, TNF-α, Oxidative stress markers	8 weeks
	Bae et al., 2009 (30)	Korea	20		Quercetin+vitamin C (166mg 133mg/capsule) + conventional treatment (Mainly hydroxychlorquine, sulfasalazine, methotexate with folate, bucillamine, NSAID, low dose steroid)	Placebo + conventional treatment (Mainly hydroxychlorquine, sulfasalazine, methotexate with folate, bucillamine, NSAID, low dose steroid)	52.1 ± 10.3		–	–	0.85(0.28,4.00)*	1.05(0.22,6.44)*	–	–	CRP, TNF-α, IL6	4 weeks
Resveratrol	Khojah et al., 2018 (31)	Egypt	50	50	Resveratrol 1000mg + conventional treatment	Placebo + conventional treatment	46.5 ± 12.3	44.2 ± 16.4	4.62 ± 0.99	4.91 ± 0.92	2.7 ± 0.7	2.9 ± 0.8	39.4 ± 11.5	43.8 ± 14.8	DAS28, CRP, ESR, TNF-α, IL6	12 weeks
Garlic extract	Moosavian et al., 2020 (32, 33)	Iran	31	31	Garlic tablets 500mg (equivalent to 2500 mg of fresh garlic, and containing 2.5 mg allicin) Bid with no changes to current medication (mainly Prednisolone, Methotroxate, Sulfasalazine)	Placebo with no changes to current medication (mainly Prednisolone, Methotroxate, Sulfasalazine)	51.06 ± 13.8	51.39 ± 10.38	4.61 ± 0.92	4.52 ± 0.78	13.44 ± 13.76	13.57 ± 14.04	23.63 ± 13.82	20.10 ± 11.74	CRP, ESR, TNF-α, Oxidative stress markers	8 weeks
Total glucosides of paeony	Zou et al., 2021 (34)	China	35	35	Total glucosides of paeony 0.6g Tid+ Hydroxychloroquine Sulfate 0.2g	Hydroxychloroquine Sulfate 0.2g	50.73 ± 7.61	50.12 ± 7.52	5.64 ± 2.09	5.68 ± 2.12	3.64 ± 1.14	3.69 ± 1.18	54.29 ± 11.93	56.48 ± 12.18	DAS28, CRP, ESR	12 weeks
	Ding et al., 2021 (35)	China	42	42	Total glucosides of paeony 0.6g Tid + Tripterygium glycosides + methotrexate	Tripterygium glycosides + methotrexate	47.45 ± 2.48	47.32 ± 2.56	5.96 ± 1.18	6.08 ± 1.24	23.21 ± 3.18	23.14 ± 3.27	–	–	DAS28, CRP, IL-6, adverse events	12 weeks
	Wu et al., 2021 (36)	China	60	60	Total glucosides of paeony 0.6g Bid + Leflunomide	Leflunomide	43.05 ± 6.12	44.02 ± 6.26	–	–	25.32 ± 4.22	25.31 ± 4.24	48.21 ± 4.42	48.24 ± 4.43	CRP, ESR, RF, IL-6	12 weeks
	Ju et al., 2019 (37)	China	60	60	Total glucosides of paeony 0.6g Bid + Methotrexate 10mg once a week+Leflunomide 10mg Qd	Methotrexate 10mg once a week+Leflunomide 10mg Qd	40.12 ± 4.37	39.81 ± 4.42	5.23 ± 0.50	5.31 ± 0.47	13.85 ± 2.73	14.25 ± 2.60	–	–	DAS28, CRP, RF, adverse events	24 weeks
	Zheng et al., 2018 (38)	China	42	40	Total glucosides of paeony 0.3g Tid for the first week and then 0.6 Tid + Methotrexate Tablets + Hydroxychloroquine	Methotrexate + Hydroxychloroquine	42.92 ± 20.65	45.78 ± 20.44	4.27 ± 1.37	4.32 ± 1.05	–	–	–	–	DAS28, CRP	48 weeks
	Yu et al., 2018A (39)	China	40	40	Total glucosides of paeony 0.6g Tid + Methotrexate + Leflunomide	Methotrexate + Leflunomide	47.18 ± 6.92	47.33 ± 6.67	7.0 ± 2.2	7.1 ± 2.3	29.8 ± 14.3	31.0 ± 15.4	56.8 ± 27.0	59.3 ± 26.1	ACR, DAS28, CRP, ESR, IL-6, RF, adverse events	36 weeks
	Yu et al., 2018B (40)	China	42	38	Total glucosides of paeony 0.6g Bid + Methotrexate + Leflunomide	Methotrexate + Leflunomide	58.61 ± 7.52	58.36 ± 7.54	–	–	32.18 ± 6.82	32.57 ± 6.85	67.21 ± 9.27	66.94 ± 9.25	ESR, CRP, RF, IL-6, TNF-α	4 weeks
	Chen et al., 2017 (41)	China	40	40	Total glucosides of paeony 0.3g Bid + Methotrexate	Methotrexate	32.8 ± 4.9	32.4 ± 5.3	–	–	–	–	–	–	Adverse events	24 weeks
	Han et al., 2016 (42)	China	42	42	Total glucosides of paeony 0.6g Tid + Leflunomide	Leflunomide	65.83 ± 7.21	66.24 ± 7.39	–	–	32.14 ± 6.83	31.86 ± 7.42	64.18 ± 12.34	63.92 ± 11.76	ESR, CRP, RF, adverse events	24 weeks
	Lu et al., 2015 (43)	China	50	50	Total glucosides of paeony 0.6g Tid + Leflunomide	Leflunomide	25~65	20~64	–	–	23.25 ± 18.30	21.95 ± 19.55	66.74 ± 38.03	70.58 ± 33.98	ESR, CRP, RF, adverse events	12 weeks
	Zheng and Xiao 2013 (44)	China	90	90	Total glucosides of paeony 0.6g Tid + Methotrexate	Methotrexate	70.13 ± 5.88	68.18 ± 5.98	4.5 ± 2.1	4.7 ± 2.2	46 ± 35	47 ± 33	33 ± 17	33 ± 19	DAS28, ESR, CRP, RF, Adverse events	12 weeks
	Zheng et al., 2014 (45)	China	34	34	Total glucosides of paeony 0.6g Tid + Methotrexate + Leflunomide	Methotrexate + Leflunomide	52 ± 6	51 ± 9	4.83 ± 2.07	4.59 ± 1.88	39 ± 12	39 ± 10	27 ± 14	29 ± 14	DAS28, ESR, CRP, Adverse events	12 weeks
	Li et al., 2011 (46)	China	60	60	Total glucosides of paeony 0.6g Tid + Tripterygium glycosides	Methotrexate	47.3 ± 22.7		–	–	–	–	64.40 ± 40.09	65.41 ± 39.49	ESR, adverse events	12 weeks
	Yu and Zhang 2010 (47)	China	40	40	Total glucosides of paeony 0.6g Tid + Leflunomide	Leflunomide	39 ± 11	46 ± 10	–	–	39 ± 24	36 ± 25	55 ± 20	59 ± 26	ESR, CRP, ACR, adverse events	24 weeks
	Shang and Liu 2009 (48)	China	39	40	Total glucosides of paeony 0.6g Tid + Methotrexate	Methotrexate	40 ± 6	39 ± 6	–	–	56 ± 23	51 ± 27	47 ± 20	48 ± 18	ESR, CRP, adverse events	12 weeks
	Wang and Liu 2008 (49)	China	40	40	Total glucosides of paeony 0.6g Tid + Vitamin D3 + Methotrexate + Leflunomide + Sodium Diclofenac	Methotrexate + Leflunomide + Sodium Diclofenac	46.5 ± 14.13	46.4 ± 11.00	–	–	45.93 ± 18.31	46.78 ± 20.01	–	–	CRP, RF	12 weeks
	Fan and Li 2006 (50)	China	34	32	Total glucosides of paeony 0.6g Tid + Methotrexate	Methotrexate	60~70	61~70	–	–	201.1 ± 132.1	177.9 ± 102	48.65 ± 34.13	49.72 ± 21.45	ESR, CRP, adverse events	24 weeks
	Shi and Yang 2006 (51)	China	35	35	Total glucosides of paeony 0.6g Tid + Methotrexate	Methotrexate	42.3 ± 12.8	44.1 ± 11.5	–	–	–	–	–	–	Adverse events	12 weeks
	Zhang et al., 2005 (52)	China	30	30	Total glucosides of paeony 0.6g Tid + Tripterygium glycosides	Tripterygium glycosides	51.3 ± 15.8	53.55 ± 13.68	–	–	143.9 ± 232	155.1 ± 181	317.82 ± 161.35	309.93 ± 156.50	ESR, CRP, RF	12 weeks
	Zhao and Liu 2006 (53)	China	40	40	Total glucosides of paeony 0.6g Tid + Leflunomide	Leflunomide	31.0 ± 8.9	30.0 ± 9.6	–	–	40.31 ± 8.64	39.58 ± 7.3	59.51 ± 15.03	60.57 ± 16.34	ESR, CRP, RF, adverse events	12 weeks
	Du and Dong 2005 (54)	China	31	30	Total glucosides of paeony 0.6g Tid + Methotrexate	Methotrexate	40.0 ± 6.4	38.0 ± 7.8	–	–	–	–	47 ± 20	48 ± 19	ESR, adverse events	12 weeks
	Chen et al., 2013 (55)	China	105	89	Total glucosides of paeony 0.6g Tid + Methotrexate + Leflunomide	Methotrexate + Leflunomide	44.6 ± 13.3		5.98 ± 1.14	6.28 ± 1.34	3.27(1.9,7.55)*	3.27(1.85,6.67)*	47.65 ± 33.45	43.06 ± 30.02	DAS28, ESR, CRP, RF, adverse events	24 weeks
	Xiang et al., 2015 (56)	China	132	136	Total glucosides of paeony 0.6g Tid + Methotrexate + Leflunomide	Methotrexate + Leflunomide	48.65 ± 12.13		5.86 ± 1.16	5.73 ± 1.14	12.40(5.83,23.40)*	13.50(4.21,25.30)*	43.00(30.00,64.00)*	39.00(27.00,68.00)*	DAS28, ESR, CRP, RF	12 weeks
Tea polyphenols	Mirtaheri et al., 2021 (57)	Iran	22	22	Stachys schtschegleevii tea + existing RA therapy	Existing RA therapy	45.6 ± 9.8	46.9 ± 10.6	3.4 ± 0.5	3.5 ± 0.6	7.59 ± 6.31	7.60 ± 7.15	–	–	DAS28, CRP, adverse events	8 weeks
	Alghadir et al., 2016 (58)	Egypt	60	60	a: Green tea extracts + infliximab v.s. Infliximab; b: Green tea extracts + exercise v.s. exercise; c: Green tea extracts v.s. infliximab + exercise		a:48.9 ± 7.1v.s.55.6 ± 12.41;b:51.6 ± 3.8v.s.52.6 ± 9.4;c:57.3 ± 11.2v.s.48.9 ± 7.1		a:5.7 ± 0.31v.s.6.6 ± 0.65;b:6.2 ± 0.37v.s.6.7 ± 0.23;c:5.4 ± 0.43v.s.5.1 ± 0.40		a:6.9 ± 0.5v.s.5.29 ± 0.7;b:6.2 ± 0.5v.s.7.1 ± 0.45;c:6.2 ± 0.52v.s.9.2 ± 0.7		a:78.5 ± 10.1v.s.68.7 ± 9.09;b:60.7 ± 11.1v.s.71.5 ± 13.1;c:65.7 ± 11.1v.s.65.5 ± 11.3		DAS28, ESR, CRP, ACR	24 weeks
Puerarin	Yang et al., 2018 (59)	China	60	59	Puerarin + existing RA therapy	Existing RA therapy	49.78-56.15	50.03-58.07	4.23(3.76-4.69)*	4.41(3.91-4.93)*	12.03(7.99-16.07)*	13.61(9.36-17.86)*	43.95(37.30-50.53)*	44.10(37.44-50.76)*	DAS28, ESR, CRP, IL-6, adverse events	24 weeks
Hesperidin	Kometani et al., 2008 (60)	Japan	9	10	Beverages containing alpha-glucosylhesperidin	Placebo beverages	26-49		–	–	–	–	–	–	Efficacy indicators, adverse events	12 weeks
Crocus sativus L. extract	Sahebari et al., 2021 (61)	Iran	28	27	Crocus sativus L. extract + Standard RA therapy	Placebo + Standard RA therapy	48.43 ± 14.69	50.80 ± 9.55	5.48 ± 1.26	5.66 ± 1	–	–	25.85 ± 18.55	34.44 ± 19.33	DAS28, ESR	12 weeks
	Hamidi et al., 2020 (62)	Iran	33	32	Crocus sativus L. extract	Placebo	51.55 ± 8.26	51.80 ± 9.62	–	–	12.00 ± 7.40	12.00 ± 12.84	29.94 ± 17.40	30.20 ± 28.19	DAS28, CRP, ESR, MDA, TAC	12 weeks
Ginger extract	Aryaeian et al., 2019 (63)	Iran	33	30	Ginger supplementation	Placebo	48.63 ± 2.38	46.67 ± 1.94	4.73 ± 0.27	4.51 ± 0.27	–	–	–	–	DAS28, other inflammatory parameters	100 weeks
Cinnamon extract	Shishehbor et al., 2018 (64)	Iran	18	18	Cinnamon extract	Placebo	44.66 ± 11.22	49.11 ± 7.45	–	–	35.33 ± 10.08	27 ± 12.92	32.88 ± 13.31	25.16 ± 17.44	DAS28, Inflammatory parameters	8 weeks
Sesamin	Helli et al., 2019 (65, 66)	Iran	22	22	Sesamin supplementation	Placebo	55.49 ± 5.98		–	–	–	–	–	–	Efficacy indicators, Inflammatory parameters, Oxidative stress markers	6 weeks
Cranberry extract	Thimóteo et al., 2019 (67)	Brazil	20	18	Cranberry juice	maintain usual diet	51-65	40-60	2.68-4.65	3.19-5.23	1.45-8.60	1.50-9.10	12-40	6-32.45	DAS28, CRP, ESR, RF	12 weeks
	Fatel et al., 2021 (68)	Brazil	20	21	Cranberry juice	maintain usual diet	47-65	43-63	2.22-3.21	2.55-4.12	1.7-7.5	2.0-9.5	10.0-27.0	11.5-39.5	DAS28, CRP, ESR, RF	12 weeks
Olive extracts and oil	Hekmatpou et al., 2020 (69)	Iran	12	48	Routine drugs + Massaging the phalanges and knees using topical olive oil	Routine drugs + massaging	40 ± 10.5		–	–	–	–	–	–	Efficacy indicators	12 weeks
	Berbert et al., 2005 (70)	Brazil	13	30	Fish oil omega-3 fatty acids + olive oil	a: placebo; b: fish oil omega-3 fatty acids	51 ± 13	a:48 ± 10;b:51 ± 13	–	–	–	–	–	–	Efficacy indicators	24 weeks
	Bitler et al., 2007 (71)	the U.S.	–	–	Freeze-dried olive vegetation water	placebo	–	–	–	–	–	–	–	–	Efficacy indicators, adverse events	8 weeks
Curcumin	Amalraj et al., 2017 (72)	India	24	12	Curcumin 250mg or 500mg	placebo	36.7 ± 10.7(250mg);38.3 ± 5.8(500mg)	39.6 ± 8.8	–	–	–	–	–	–	DAS28, ESR, CRP, RF, adverse events	12 weeks
	Pourhabibi-Zarandi et al., 2022 (73)	Iran	22	22	Curcumin 500 mg	placebo	50.68 ± 9.93	50.36 ± 9.70	–	–	20.40 ± 14.30	20.34 ± 10.66	29.09 ± 8.74	24.32 ± 6.99	ESR, CRP	8 weeks
	Javadi et al., 2019 (74)	Iran	24	25	Curcumin nanomicelles 40mg Tid	Placebo	53.71 ± 2.75	56.28 ± 2.5	3.75 ± 1.01	3.45 ± 0.95	–	–	–	–	DAS28, ESR, adverse events	12 weeks
	Jacob et al., 2018 (75)	India	16	8	a: Curcumin 250mg; b: 500mg	placebo	18-65	18-65	–	–	a:9.7 ± 1.7;b:11.4 ± 2.2	9.9 ± 1.6	a:179.12 ± 13.85;b:189.37 ± 8.01	183.75 ± 11.97	DAS28, ESR, CRP, RF	12 weeks
	Chandran and Goel 2012 (76)	India	30	15	a: Curcumin 500 mg; b: Curcumin 500 mg+diclofenac sodium 50 mg	Diclofenac sodium 50 mg	a:47.8 ± 8.60;b:47 ± 16.22	48.87 ± 10.78	a:6.40 ± 0.73;b:6.44 ± 0.51	6.72 ± 0.87	a:5.34 ± 4.12;b:9.11 ± 9.93	3.3 ± 2.4	a:28 ± 23.7;b:28.75 ± 20.9	27.08 ± 17.1	DAS28, ESR, CRP, adverse events	8 weeks

*The data is shown as M (P25, P75).

### 3.3 Risk of bias assessments

**Figure 2:**
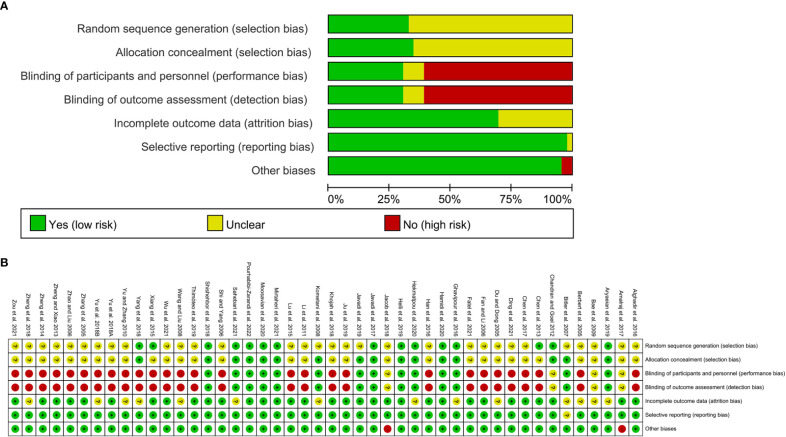
**(A)** risk of bias graph; **(B)** risk of bias summary.

The summary and graph of risk of bias ware shown in **Figure 2**.

### 3.4 Pomegranate extract for RA

Only Ghavipour et al., 2016 from Iran reported the results of Pomegranate extract in the treatment of RA involving 30 participants in Pomegranate extract group and 25 in control group (27). The control group received cellulose capsules as a placebo. They found that compared with placebo, pomegranate extract reduced DAS28 scores, improved joint swelling and tenderness, decreased ESR levels, and increased gluthation peroxidase (GPx) concentrations (27). This suggests that Pomegranate extract may play a role in the treatment of RA through anti-inflammatory and anti-oxidation. However, its lowering effect on MMP3, CRP and MDA has not yet been found.

### 3.5 Quercetin for RA

Javadi et al., 2017 (28, 29) from Iran and Bae et al., 2009 (29) from Korea reported the results of quercetin in the treatment of RA involving 120 participants. Because the results of the 2 RCTs could not be combined, only a systematic review was performed. Javadi et al., 2017 found that DAS-28 decreased and serum TNF-α levels were significantly reduced after quercetin intervention compared to placebo (28, 29). However, Bae et al., 2009 showed no significant differences in serum proinflammatory cytokines (TNF-α and IL-1β) and CRP levels after quercetin treatment compared with placebo (30). This suggests that the effect of quercetin in the treatment of RA needs more research.

### 3.6 Resveratrol for RA

Khojah et al., 2018 from Egypt reported the results of resveratrol in the treatment of RA involving 100 participants (50 in each group) (31). They found that resveratrol treatment reduced swollen and tender joint counts, decreased DAS28, and decreased serum CRP, ESR, hypocarboxylated osteocalcin, matrix metalloproteinase (MMP)3, TNF-α, and IL-6.

### 3.7 Garlic extract for RA

Moosavian et al., 2020 from Iran reported the results of garlic extract in the treatment of RA involving 62 participants (31 in each group) (32). They found that the Garlic extract intervention improved antioxidant levels (increased TAC, decreased MDA), improved quality of life, and improved inflammation (decreased CRP and TNF-α), decreased joint pain and tenderness, and decreased DAS28 compared with the placebo group.

### 3.8 Total glucosides of paeony for RA

A total of 23 RCTs reported total glucosides of paeony in the treatment of RA, efficacy indicators, inflammatory parameters and adverse event outcomes were reported, so a meta-analysis was performed.

#### 3.8.1 Efficacy indicators

Efficacy indicators include DAS28, ACR20, ACR50 and ACR70.

**Figure 3:**
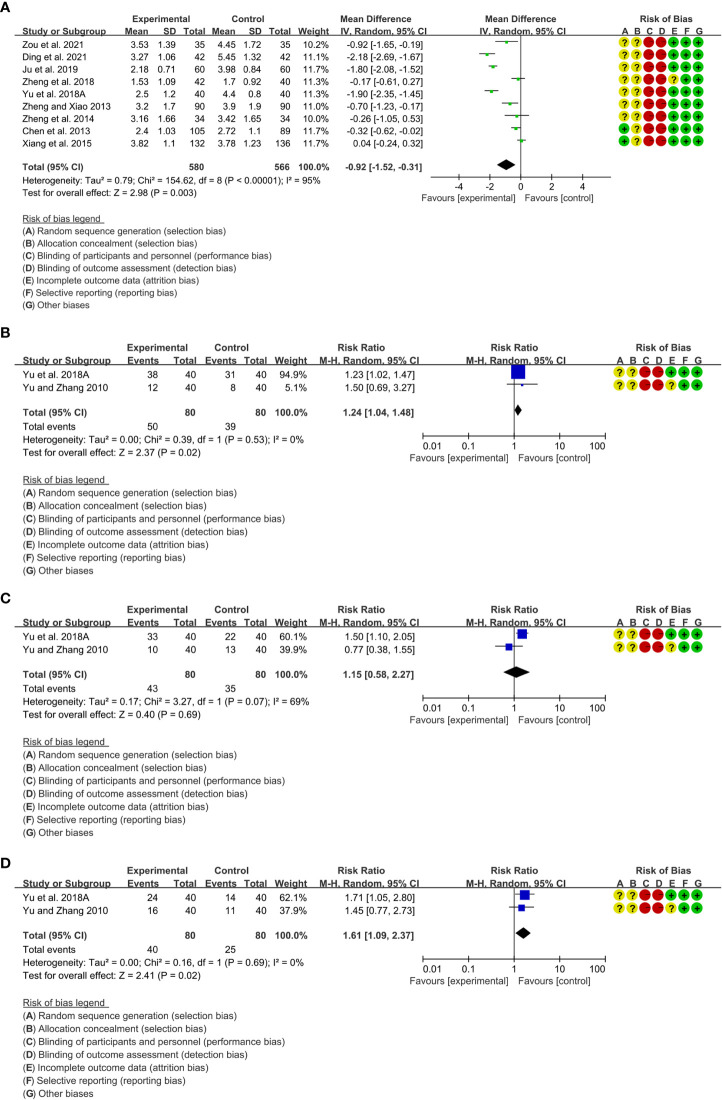
Efficacy indicators of Total glucosides of paeony **(A)** DAS28; **(B)** ACR20; **(C)** ACR50; **(D)** ACR70.

1. A total of 9 RCTs reported DAS28. Heterogeneity analysis suggested high heterogeneity among RCTs (I2 = 95%, P<0.00001), and a random-effects model was used. The results showed that compared with the control group, the DAS28 of the total glucosides of paeony was lower [WMD=-0.92 95%CI (-1.52, -0.31), P=0.003] (**Figure 3A**).
2. Only 2 RCTs reported ACR20. Heterogeneity analysis suggested low heterogeneity among RCTs (I2 = 0%, P=0.53), and a random-effects model was used. The results showed that compared with the control group, the ACR20 of the total glucosides of paeony was lower [RR=1.24 95%CI(1.04, 1.48), P=0.02] (**Figure 3B**).
3. Only 2 RCTs reported ACR50. Heterogeneity analysis suggested high heterogeneity among RCTs (I2 = 69%, P=0.07), and a random-effects model was used. The results showed that the ACR50 between two groups was of no statistical significance [RR=1.15 95%CI(0.58, 2.27), P=0.69] (**Figure 3C**).
4. Only 2 RCTs reported ACR70. Heterogeneity analysis suggested low heterogeneity among RCTs (I2 = 0%, P=0.53), and a random-effects model was used. The results showed that compared with the control group, the ACR70 of the total glucosides of paeony was lower [RR=1.61 95%CI(1.09, 2.37), P=0.02] (**Figure 3D**).

#### 3.8.2 Inflammatory parameters

Inflammatory parameters include CRP, ESR, RF, IL-6 and TNF-α.

**Figure 4:**
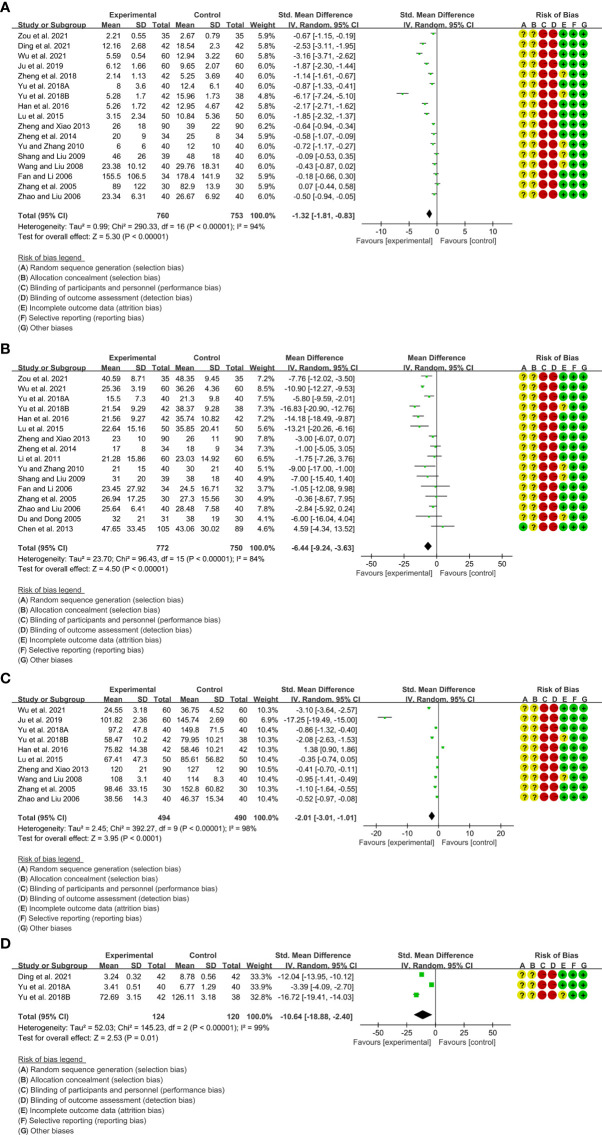
Inflammatory parameters of Total glucosides of paeony **(A)** CRP; **(B)** ESR; **(C)** RF; **(D)** IL-6.

1. A total of 17 RCTs reported CRP. Heterogeneity analysis suggested high heterogeneity among RCTs (I2 = 94%, P<0.00001), and a random-effects model was used. The results showed that compared with the control group, the CRP of the total glucosides of paeony was lower [SMD=-1.32 95%CI (-1.81, -0.83), P<0.00001] (**Figure 4A**).
2. A total of 16 RCTs reported ESR. Heterogeneity analysis suggested high heterogeneity among RCTs (I2 = 84%, P<0.00001), and a random-effects model was used. The results showed that compared with the control group, the ESR of the total glucosides of paeony was lower [WMD=-6.44 95%CI (-9.24, -3.63), P<0.00001] (**Figure 4B**).
3. A total of 10 RCTs reported RF. Heterogeneity analysis suggested high heterogeneity among RCTs (I2 = 98%, P<0.00001), and a random-effects model was used. The results showed that compared with the control group, the RF of the total glucosides of paeony was lower [SMD=-2.01 95%CI (-3.01, -1.01), P<0.0001] (**Figure 4C**).
4. Only 3 RCTs reported IL-6. Heterogeneity analysis suggested low heterogeneity among RCTs (I2 = 99%, P<0.00001), and a random-effects model was used. The results showed that compared with the control group, the IL-6 of the total glucosides of paeony was lower [WMD=-10.64 95%CI(1.08, 2.36), P=0.02] (**Figure 4D**).
5. Only Yu et al., 2018B reported TNF-α. They found that the TNF-α in total glucosides of paeony group was lower than that in control group (P<0.05).

#### 3.8.3 Adverse events

**Figure 5:**
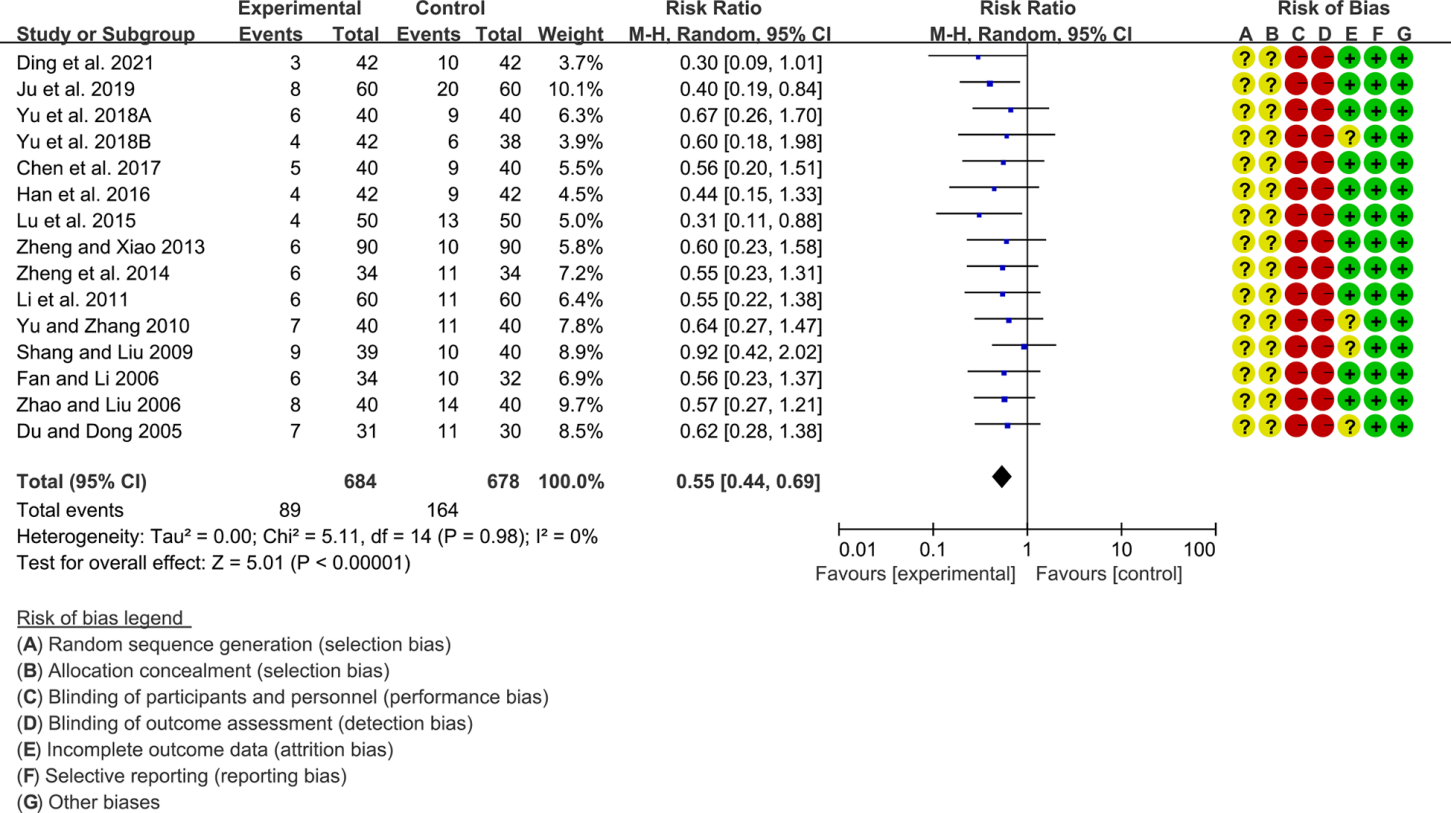
Adverse events in Total glucosides of paeony.

A total of 15 RCTs reported adverse events. Heterogeneity analysis suggested low heterogeneity among RCTs (I2 = 0%, P=0.98), and a random-effects model was used. The results showed that compared with the control group, the adverse events of the total glucosides of paeony were lower [SMD=0.55 95%CI (0.44, 0.69), P<0.00001] (**Figure 5**).

### 3.9 Tea polyphenols for RA

Only 2 RCTs reported tea polyphenols in the treatment of RA, efficacy indicators, inflammatory parameters and adverse event outcomes were reported, so a meta-analysis was performed.

#### 3.9.1 Efficacy indicators

Efficacy indicators include DAS28, ACR20, ACR50 and ACR70.

**Figure 6:**
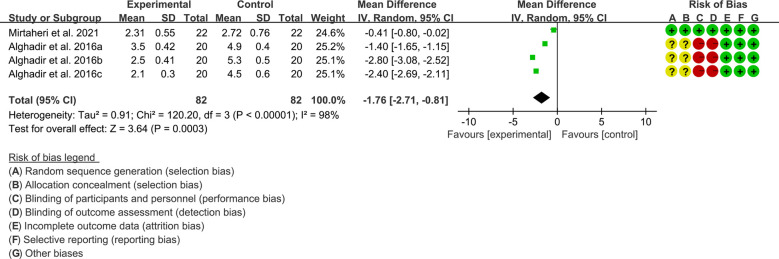
DAS28 in Tea polyphenols.

1. Both 2 RCTs reported DAS28. Heterogeneity analysis suggested low heterogeneity among RCTs (I2 = 98%, P<0.00001), and a random-effects model was used. The results showed that compared with the control group, the DAS28 of the tea polyphenols were lower [WMD=-1.76 95%CI (-2.71, -0.81), P=0.0003] (**Figure 6**).
2. Only Alghadir et al., 2016 reported ACR20, ACR50 and ACR70 and they found that after the addition of tea polyphenols, the ACR20, ACR50 and ACR70 was higher than control group (58).

#### 3.9.2 Inflammatory parameters

Inflammatory parameters include CRP and ESR.

**Figure 7:**
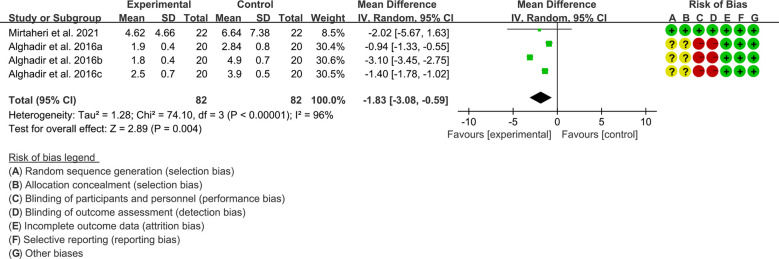
CRP in Tea polyphenols.

1. Both 2 RCTs reported CRP. Heterogeneity analysis suggested high heterogeneity among RCTs (I2 = 96%, P<0.00001), and a random-effects model was used. The results showed that compared with the control group, the CRP of the tea polyphenols was lower [WMD=-1.83 95%CI (-3.08, -0.59), P=0.004] (**Figure 7**).
2. Only Alghadir et al., 2016 reported ESR and they found that after the addition of tea polyphenols, the ESR was higher than control group.

#### 3.9.3 Adverse events

Mirtaheri et al., 2021 showed that no adverse effects of treatment were recorded in the tea polyphenol group and the control group (57). Alghadir et al., 2016 did not report any adverse reactions, possibly because they were not monitored (58).

### 3.10 Puerarin for RA

Yang et al., 2018 from China reported Puerarin in the treatment of RA, involving 60 participants in experiments group and 59 participants in control group (59). They found that DAS28, ESR, CRP, IL-6 was lower after Puerarin intervention. They also found no significant difference in adverse event rates between the two groups.

### 3.11 Hesperidin for RA

Kometani et al., 2008 from Japan reported Hesperidin in the treatment of RA, involving 9 participants in experiments group and 10 participants in control group (60). They found that RA symptoms improved (3 out of 9 patients) after the patients drank the Hesperidin-containing beverage, compared with only 1 improvement in 10 patients in the control group.

### 3.12 Crocus sativus L. extract for RA

Only 2 RCTs reported Crocus sativus L. extract in the treatment of RA, efficacy indicators, inflammatory parameters, oxidative stress markers and adverse event outcomes were reported, so a meta-analysis was performed.

#### 3.12.1 Efficacy indicators

**Figure 8:**
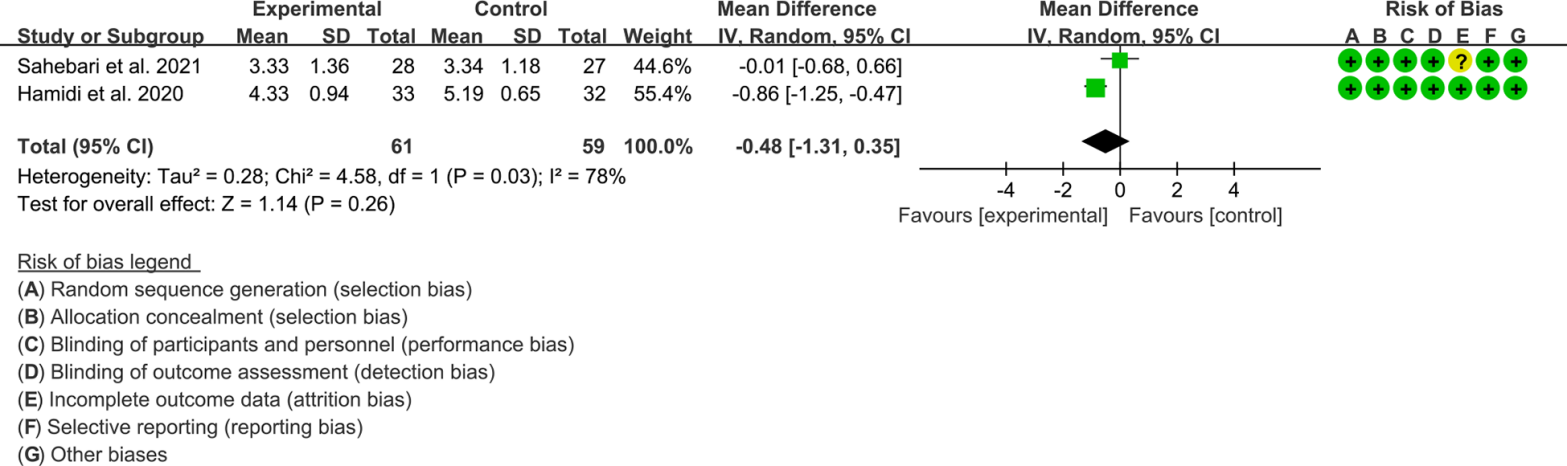
DAS28 in Crocus sativus L.

Both 2 RCTs reported DAS28. Heterogeneity analysis suggested high heterogeneity among RCTs (I2 = 78%, P=0.03), and a random-effects model was used. The results showed that the difference of DAS29 between two groups was of no statistical significance [WMD=–0.48 95%CI (-1.31, 0.35), P=0.26] (**Figure 8**).

#### 3.12.2 Inflammatory parameters

Inflammatory parameters include CRP and ESR.

**Figure 9:**
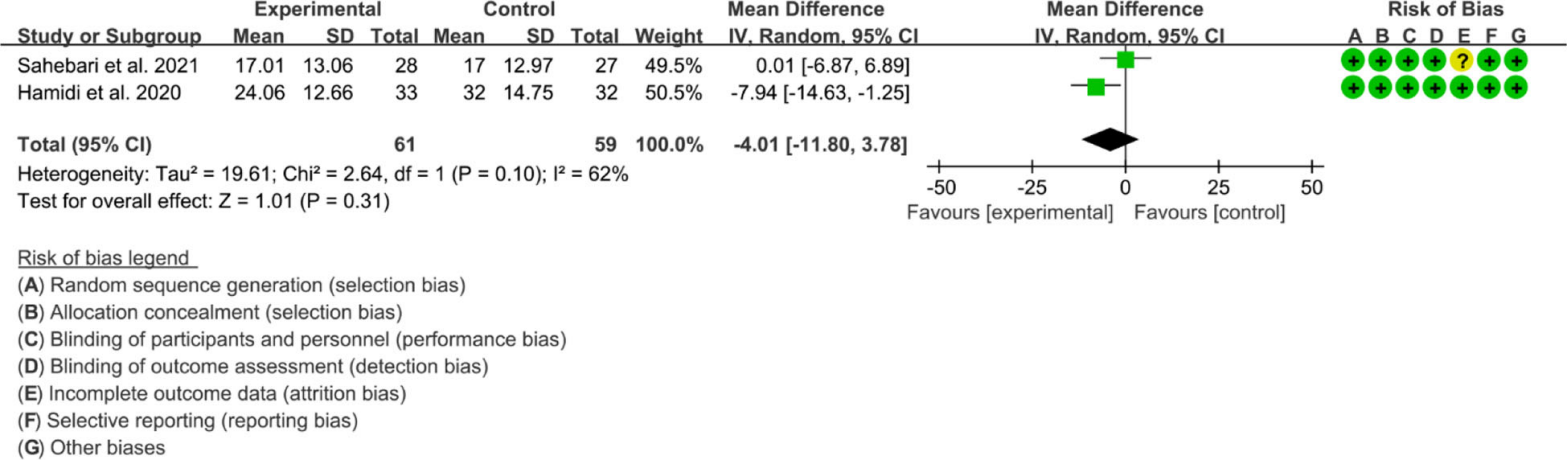
ESR in Crocus sativus L.

1. Only Hamidi et al., 2020 reported CRP. They found that the CRP decreased after Crocus sativus L. extract intervention.
2. Both 2 RCTs reported ESR. Heterogeneity analysis suggested high heterogeneity among RCTs (I2 = 62%, P=0.10), and a random-effects model was used. The results showed that the difference of ESR between two groups was of no statistical significance [WMD=-4.01 95%CI (-11. 80, 3.78), P=0.31] (**Figure 9**).

#### 3.12.3 Oxidative stress markers

Only Hamidi et al., 2020 reported MDA and TAC. They found that both MDA and TAC increased after the intervention, but there was no significant difference compared with the control group (P>0.05) (62).

#### 3.12.4 Adverse events

Adverse events were not reported in either RCTs, probably because they did not monitor for adverse events.

### 3.13 Ginger extract for RA

Only Aryaeian et al., 2019 reported Ginger extract in the treatment of RA. They found that DAS29 was reduced after the ginger intervention compared to the control group. Regarding inflammatory parameters, they found that peroxidase-activated proliferative receptor (PPAR)-γ gene expression was significantly increased in the ginger group and the control group, but there was no significant difference between the two groups (63).

### 3.14 Cinnamon extract for RA

Only Shishehbor et al., 2018 reported Cinnamon extract in the treatment of RA. They found that DAS28 decreased, the number of tender and swollen joints decreased, and serum CRP and TNF-α levels were significantly reduced after the cinnamon extract intervention (64). However, no difference in ESR between the two groups was observed.

### 3.15 Sesamin for RA

Only Helli et al., 2019 reported Sesamin in the treatment of RA. They found that compared with the placebo group, after sesamin intervention, the joint pain was relieved, the number of tender joints was reduced, and the serum hs-CRP, TNF-α and cyclooxysynthase (COX)-2 levels were significantly reduced (65, 66).

### 3.16 Cranberry extract for RA

Only 2 RCTs reported Cranberry extract in the treatment of RA. Thimóteo et al., 2019 found that after 12 weeks of cranberry extract treatment, DAS28 was decreased, but inflammatory parameters (such as CRP, ESR, RF) were not significantly different compared with the control group (67). Fatel et al., 2021 found that ESR, CRP and DAS28 were both decreased after fish oil + cranberry extract treatment compared with the control group and the treatment group with fish oil alone (68).

### 3.17 Olive extracts and oil for RA

Only 2 RCTs reported olive oil and 1 RCTs reported olive extracts in the treatment of RA. Hekmatpou et al., 2020 found that after adding olive oil massage, DAS28 decreased, joint pain was relieved, and the number of painful joints and the number of swollen joints decreased (69). Berbert et al., 2005 also found relief of joint pain and decreased duration of morning stiffness, episodes of fatigue, and Ritchie joint index in patients treated with olive oil (70).

Bitler et al., 2007 showed that after treatment with frozen-dried olive vegetation water, RA patients had reduced pain and improved activities of daily living, significantly decreased homocysteine, and was well tolerated by frozen-dried olive vegetation water (71).

### 3.18 Curcumin for RA

A total of 5 RCTs reported curcumin in the treatment of RA, efficacy indicators, inflammatory parameters and adverse event outcomes were reported, so a meta-analysis was performed.

#### 3.18.1 Efficacy indicators

**Figure 10:**
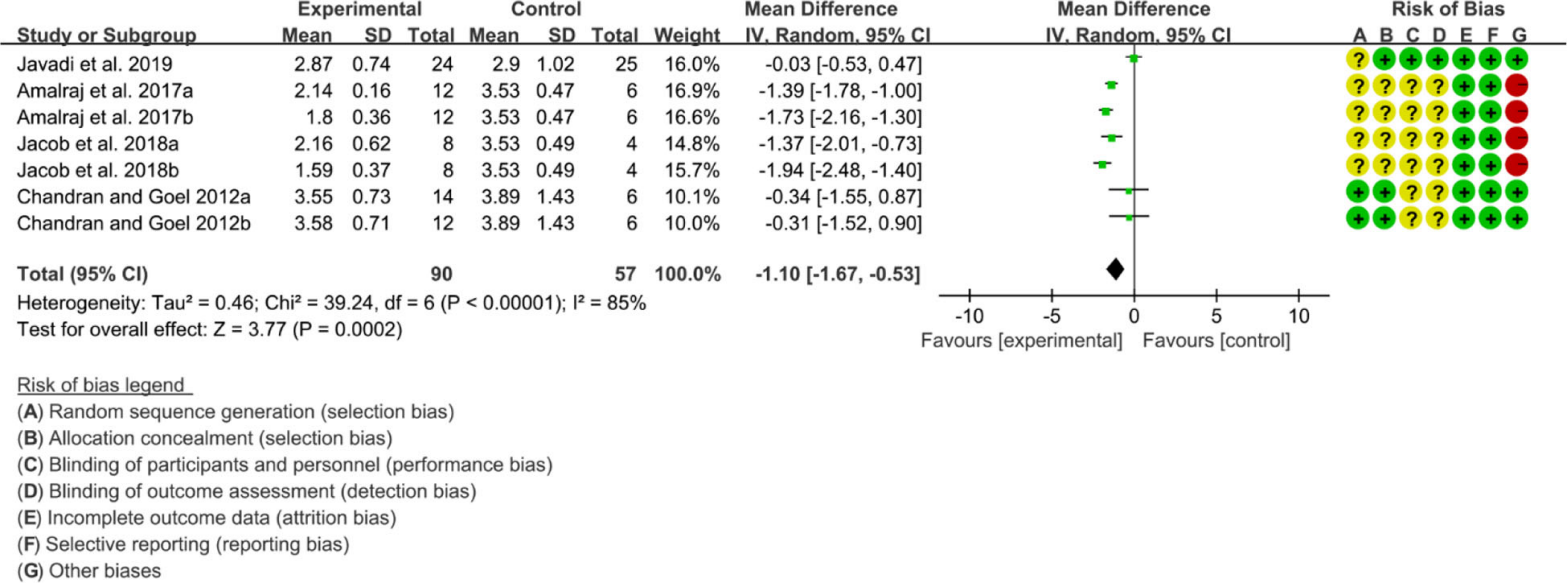
DAS28 in curcumin.

Four (4) RCTs reported DAS28. Heterogeneity analysis suggested high heterogeneity among RCTs (I2 = 85%, P<0.00001), and a random-effects model was used. The results showed that compared with the control group, the DAS29 in curcumin was lower [WMD=-1.10 95%CI (-1.67, -0.53), P=0.0002] (**Figure 10**).

#### 3.18.2 Inflammatory parameters

Inflammatory parameters include CRP, ESR and RF.

**Figure 11:**
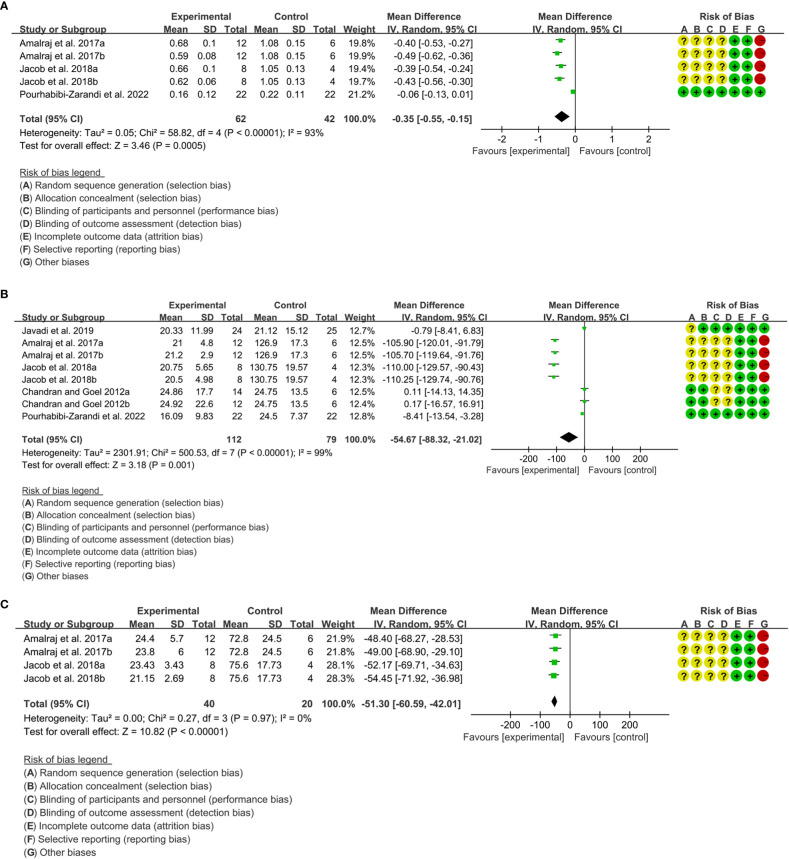
Inflammatory parameters of curcumin **(A)** CRP; **(B)** ESR; **(C)** RF.

1. Three (3) RCTs reported CRP. Heterogeneity analysis suggested high heterogeneity among RCTs (I2 = 93%, P<0.00001), and a random-effects model was used. The results showed that compared with the control group, the CRP in curcumin was lower [WMD=-0.35 95%CI (-0.55, -0.15), P=0.0005] (**Figure 11A**).
2. Five (5) RCTs reported ESR. Heterogeneity analysis suggested high heterogeneity among RCTs (I2 = 99%, P<0.00001), and a random-effects model was used. The results showed that compared with the control group, the ESR in curcumin was lower [WMD=-54.67 95%CI (-88.32, -21.02), P=0.001] (**Figure 11B**).
3. Two (2) RCTs reported RF. Heterogeneity analysis suggested high heterogeneity among RCTs (I2 = 0%, P=0.97), and a random-effects model was used. The results showed that compared with the control group, the RF in curcumin was lower [WMD=-51.30 95%CI (-60.59, -42.01), P<0.00001] (**Figure 11C**).

#### 3.18.3 Adverse events

**Figure 12:**
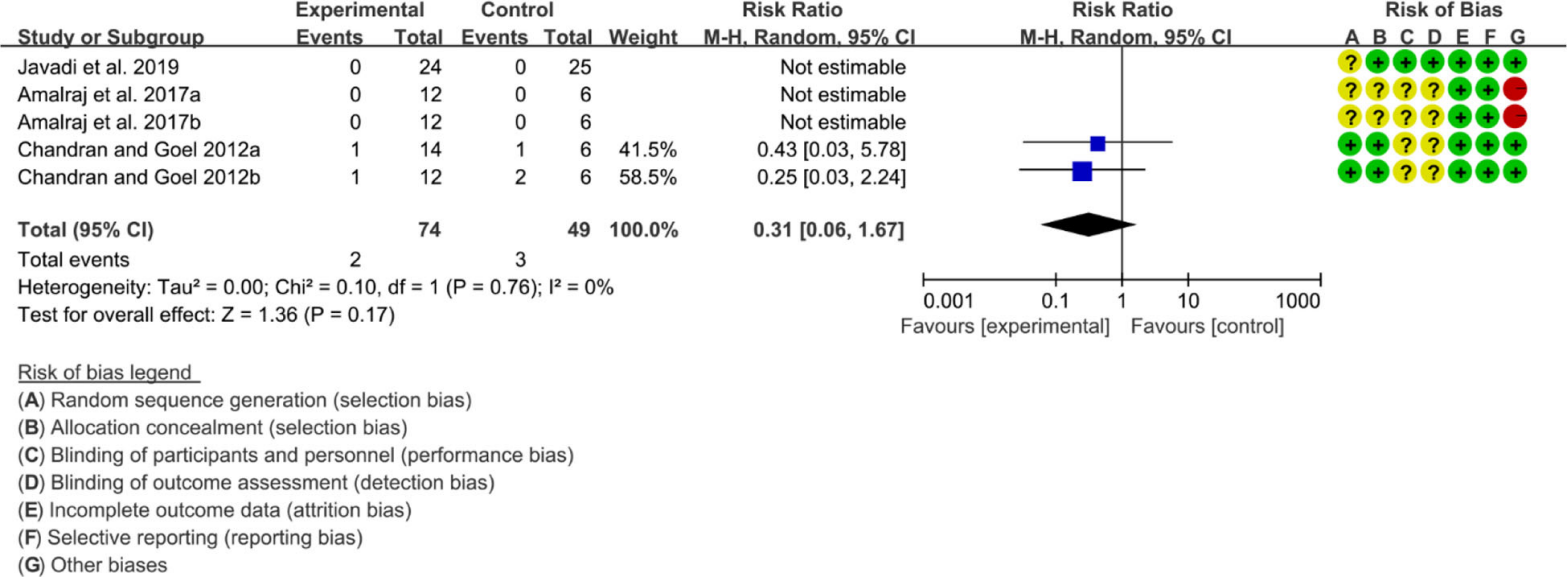
Adverse events.

Three (3) RCTs reported adverse events. Heterogeneity analysis suggested high heterogeneity among RCTs (I2 = 0%, P=0.76), and a random-effects model was used. The results showed that the addition of curcumin may not increase the incidence of adverse events [RR=0.31 95%CI (0.06, 1.67), P=0.17] (**Figure 12**).

### 3.19 Further analysis for outcomes

#### 3.19.1 Subgroup analysis

The number of RCTs for total glucosides of paeony and curcumin exceeded 4, so a subgroup analysis was performed according to the duration and dosage (**Tables 2**, **3**).

1. Total glucosides of paeony: The difference from the results above is that although the summary results of DAS28 were statistically significant, subgroup analysis showed no statistical difference in DAS28 at each time point. The ESR results also showed lower ESR when receiving ≤12 weeks of treatment, while the effect of receiving 24 weeks of treatment was similar to that of the control group (Because there is only one RCT result at 36 weeks, it is not clear that the treatment effect of 36 weeks is better than that of the control group). In addition, for RF, patients receiving total glucosides of paeony twice a day had lower RF than controls, while patients receiving total glucosides of paeony three times a day had similar RF to controls.
2. Curcumin: The difference from the results above is that after subgroup analysis of doses, there was no statistically significant difference in ESR between the experimental and control groups regardless of doses ≤ 250 mg or 500 mg. And after subgroup analysis of time. At 8 weeks of curcumin treatment, the ESR and CRP of the experimental group were not statistically different from those of the control group, and the statistical difference only appeared at 12 weeks.

**Table 2:** Subgroup analysis results of Total glucosides of paeony.

Outcomes	Subgroup	Overall effect			Heterogeneity test		Statistical method	Studies (N)	Sample size (N)	Figure
		Effect	95%CI	P	I^2^ (%)	P(Q)				
DAS28	12 weeks	WMD=-0.80	[-1.68, 0.07]	0.07	93.09	<0.001	Random	5	670	**Figure S1**
	24 weeks	WMD=-1.06	[-2.51, 0.39]	0.15	98.00	<0.001	Random	2	314
	≥36 weeks	WMD=-1.03	[-2.73, 0.66]	0.23	96.61	<0.001	Random	2	162
ESR	≤12 weeks	WMD=-6.53	[-9.92, -3.13]	<0.001	87.17	<0.001	Random	11	1018	**Figure S2**
	24 weeks	WMD=-5.57	[-14.50, 3.37]	0.22	81.66	<0.001	Random	4	424
	36 weeks	WMD=-5.80	[-9.59, -2.01]	<0.001	–	–	Random	1	80
CRP	≤12 weeks	SMD=-1.44	[-2.14, -0.73]	<0.001	95.90	<0.001	Random	11	1001	**Figure S3**
	24 weeks	SMD=-1.23	[-2.13, -0.33]	<0.001	92.98	<0.001	Random	4	350
	≥36 weeks	SMD=-1.00	[-1.33, -0.67]	0.00	0.00	0.42	Random	2	162
RF	≤12 weeks	SMD=-1.20	[-1.89, -0.50]	<0.001	94.23	<0.001	Random	7	700	**Figure S4**
	≥ 24 weeks	SMD=-5.26	[-9.62, -0.89]	0.02	99.26	<0.001	Random	3	284
Adverse events	≤12 weeks	RR=0.56	[0.42, 0.77]	<0.001	0.00	0.86	Random	9	852	**Figure S5**
	≥24 weeks	RR=0.53	[0.37, 0.76]	<0.001	0.00	0.95	Random	6	510
DAS28	Twice a day	WMD=-1.8	[-2.08, -1.52]	0.00	–	–	Random	1	120	**Figure S6**
	Three times a day	WMD=-0.80	[-1.38, -0.21]	<0.001	92.85	<0.001	Random	8	1026
ESR	Twice a day	WMD=-13.54	[-19.32, -7.77]	<0.001	86.33	0.00684	Random	2	200	**Figure S7**
	Three times a day	WMD=-5.13	[-7.67, -2.58]	<0.001	67.79	0.000121	Random	14	1322
CRP	Twice a day	SMD=-3.67	[-5.56, -1.77]	<0.001	96.50	<0.001	Random	3	320	**Figure S8**
	Three times a day	SMD=-0.87	[-1.23, -0.50]	<0.001	88.58	<0.001	Random	14	1193
RF	Twice a day	SMD=-7.15	[-11.07, -3.23]	<0.001	98.79	<0.001	Random	3	320	**Figure S9**
	Three times a day	SMD=-0.40	[-0.94, 0.15]	0.15	91.30	<0.001	Random	7	664
Adverse events	Twice a day	RR=0.48	[0.28, 0.81]	<0.001	0.00	0.80	Random	3	280	**Figure S10**
	Three times a day	RR=0.57	[0.44, 0.74]	<0.001	0.00	0.96	Random	12	1082

**Table 3:** Subgroup analysis results of curcumin.

Outcomes	Subgroup	Overall effect			Heterogeneity test		Statistical method	Studies (N)	Sample size (N)	Figure
		Effect	95%CI	P	I^2^ (%)	P(Q)				
DAS28	≤250mg	MD=-0.93	[-1.83, -0.02]	0.045	89.86	<0.001	Random	3	79	**Figure S11**
	500mg	MD=-1.30	[-1.99, -0.61]	<0.001	71.11	0.02	Random	4	68
ESR	≤250mg	MD=-71.94	[-153.31, 9.42]	0.083	99.13	<0.001	Random	3	79	**Figure S12**
	500mg	MD=-44.53	[-89.09, 0.02]	0.050	98.46	<0.001	Random	5	112
CRP	250mg	MD=-0.40	[-0.49, -0.30]	<0.001	0.00	0.92	Random	2	30	**Figure S13**
	500mg	MD=-0.32	[-0.63, -0.02]	0.037	95.76	<0.001	Random	3	74
RF	250mg	MD=-50.52	[-63.67, -37.37]	<0.001	0.00	0.78	Random	2	30	**Figure S14**
	500mg	MD=-52.08	[-65.21, -38.95]	<0.001	0.00	0.69	Random	2	30
DAS28	8 weeks	MD=-0.33	[-1.18, 0.53]	0.46	0.00	0.97	Random	2	38	**Figure S15a**
	12 weeks	MD=-1.29	[-1.93, -0.65]	<0.001	88.40	<0.001	Random	5	109
ESR	8 weeks	MD=-4.51	[-9.38, 0.36]	0.069	19.28	0.29	Random	4	131	**Figure S15b**
	12 weeks	MD=-107.27	[-115.32, -99.22]	<0.001	0.00	0.97	Random	4	60
CRP	8 weeks	MD=-0.06	[-0.13, 0.01]	0.084	–	–	Random	1	44	**Figure S15c**
	12 weeks	MD=-0.43	[-0.50, -0.36]	<0.001	0.00	0.72	Random	4	60

#### 3.19.2 Sensitivity analysis

**Figure 13:**
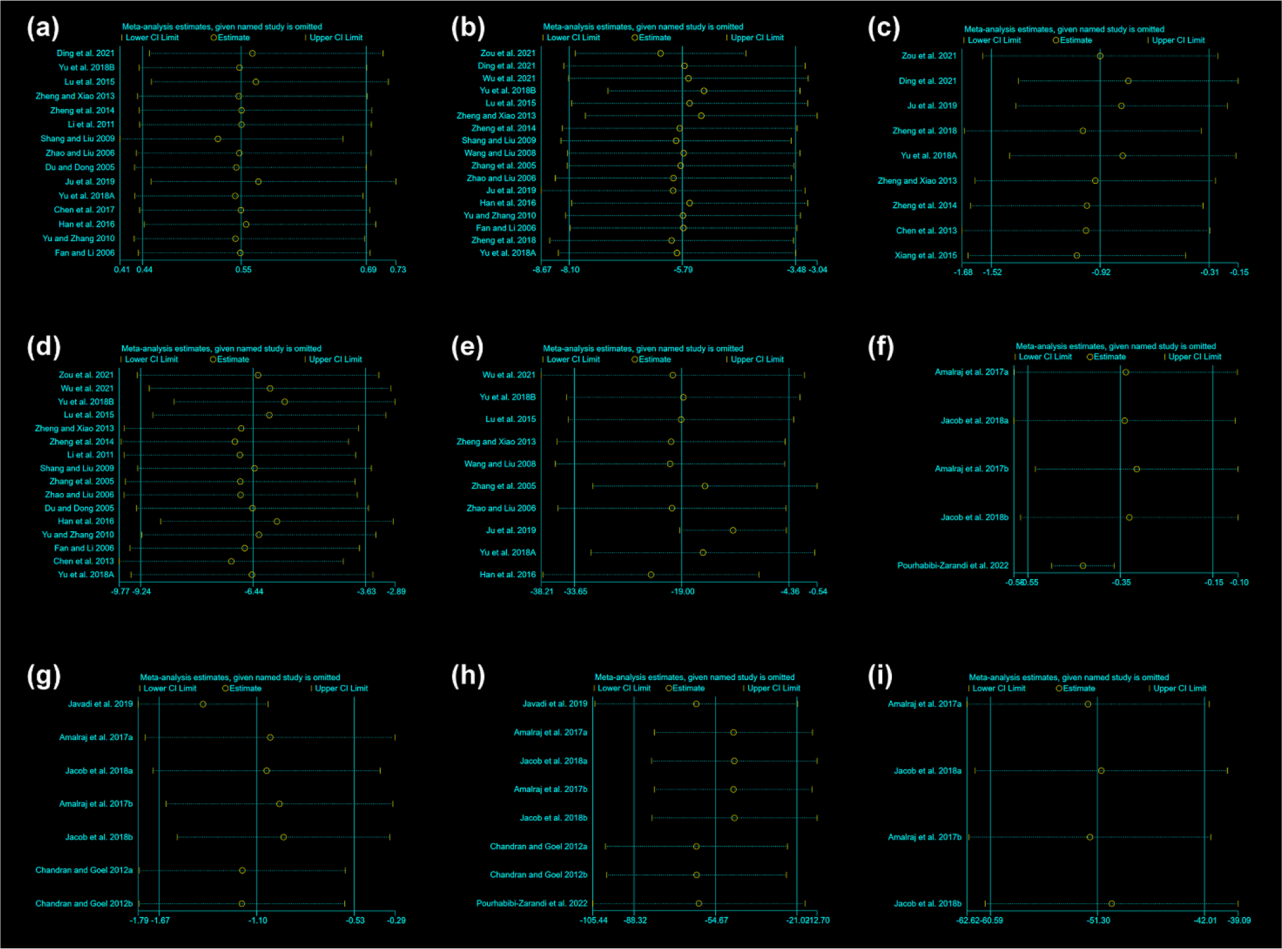
Sensitivity Analysis results **(A)** adverse events of total glucosides of paeony; **(B)** CRP of total glucosides of paeony; **(C)** DAS28 of total glucosides of paeony; **(D)** ESR of total glucosides of paeony; **(E)** RF of total glucosides of paeony; **(F)** CRP of curcumin; **(G)** DAS28 of curcumin; **(H)** ESR of curcumin; **(I)** RF of curcumin.

The sensitivity analysis was performed for total glucosides of paeony and curcumin (**Figure 13**). The sensitivity analysis showed that no matter which RCTs were removed, it had little effect on the overall results, suggesting that the results were stable.

#### 3.19.3 Publication bias analysis

**Figure 14:**
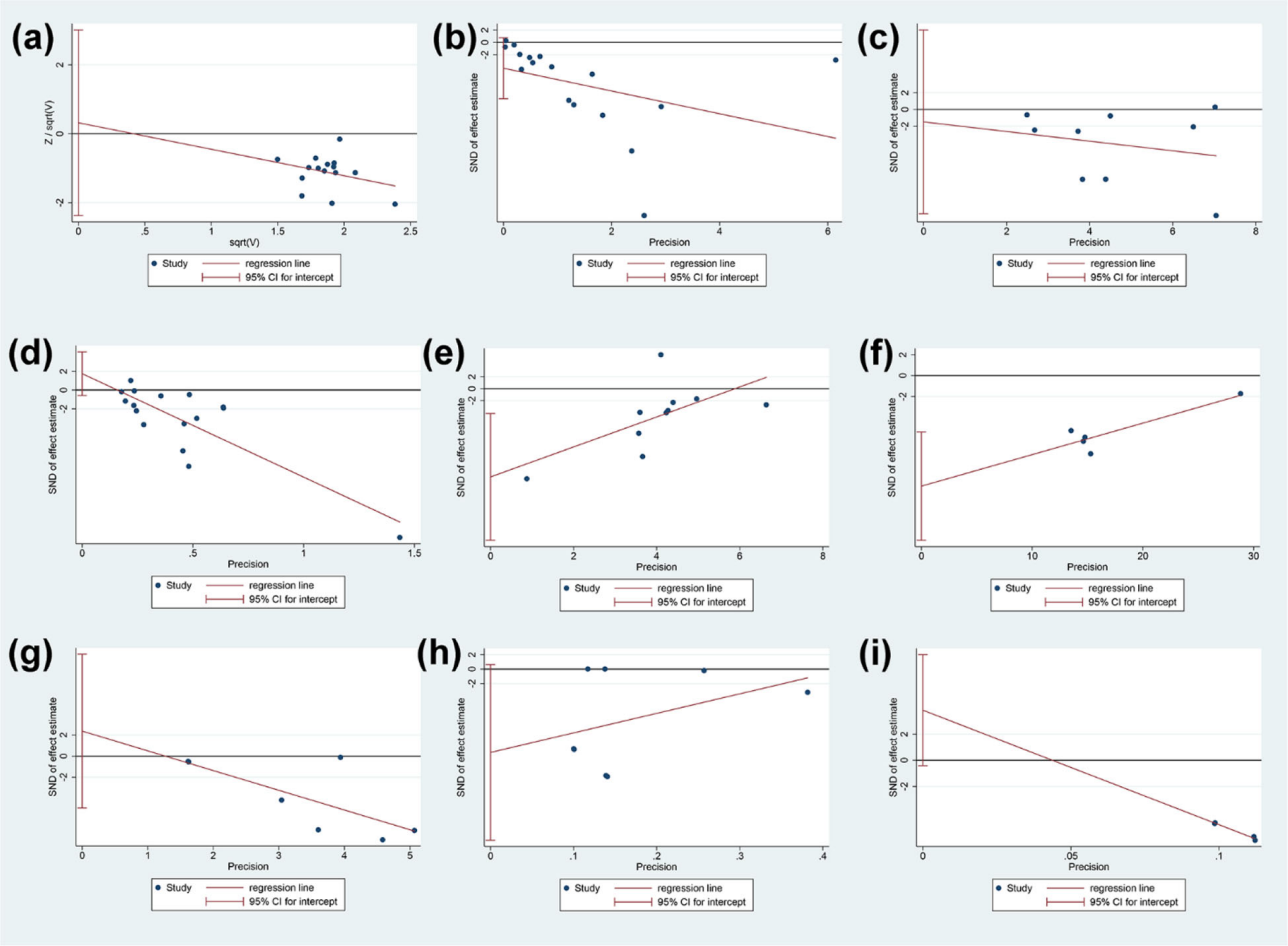
Publication bias results **(A)** adverse events of total glucosides of paeony; **(B)** CRP of total glucosides of paeony; **(C)** DAS28 of total glucosides of paeony; **(D)** ESR of total glucosides of paeony; **(E)** RF of total glucosides of paeony; **(F)** CRP of curcumin; **(G)** DAS28 of curcumin; **(H)** ESR of curcumin; **(I)** RF of curcumin.

The publication bias was performed for total glucosides of paeony and curcumin (**Figure 14**). (1) adverse events of total glucosides of paeony: it may not have publication bias (P=0.802); (2) CRP of total glucosides of paeony: it may have publication bias (P=0.091); (3) DAS28 of total glucosides of paeony: it may not have publication bias (P=0.759); (4) ESR of total glucosides of paeony: it may not have publication bias (P=0.130); (5) RF of total glucosides of paeony: it may have publication bias (P=0.012); (6) CRP of curcumin: it may have publication bias (P=0.007); (7) DAS28 of curcumin: it may not have publication bias (P=0.441); (8) ESR of curcumin: it may have publication bias (P=0.059); (9)RF of curcumin: it may have publication bias (P=0.060) (**Figure 14**).

#### 3.19.4 Meta-regression results

**Figure 15:**
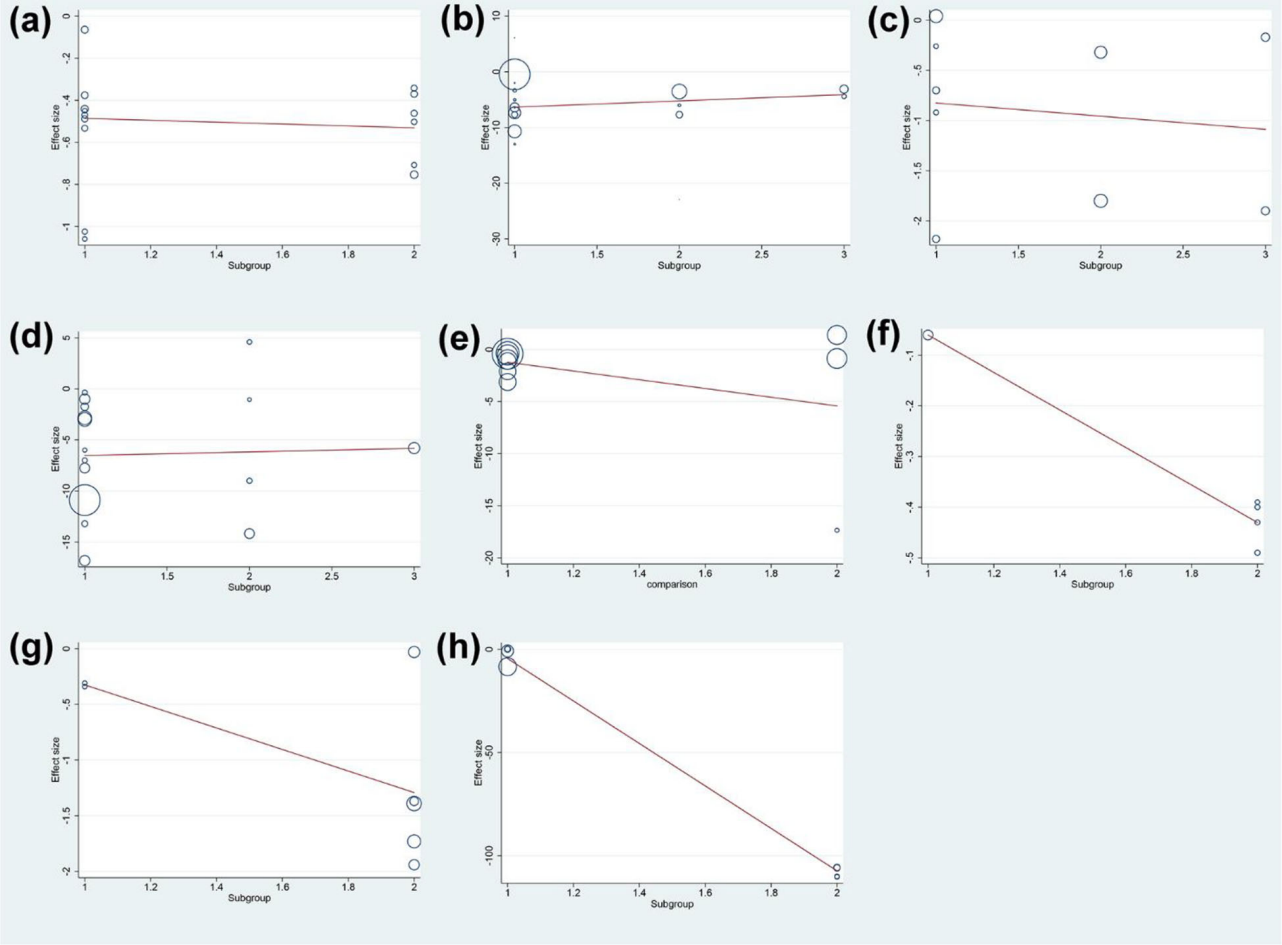
Meta-regression Results of the duration of intervention **(A)** adverse events of total glucosides of paeony; **(B)** CRP of total glucosides of paeony; **(C)** DAS28 of total glucosides of paeony; **(D)** ESR of total glucosides of paeony; **(E)** RF of total glucosides of paeony; **(F)** CRP of curcumin; **(G)** DAS28 of curcumin; **(H)** ESR of curcumin.

The results of meta-regression showed that the duration of intervention was not the source of heterogeneity in DAS28 (P=0.065), ESR (P=0.890), CRP (P=0.324), RF (P=0.271) and adverse events (P=0.859) of total glucosides of paeony. The duration of intervention was also not the source of heterogeneity in DAS28 (P=0.228) of curcumin. However, the duration of intervention may be the source of heterogeneity in ESR (P<0.0001) and CRP (P=0.005) of curcumin (**Figure 15**).

**Figure 16:**
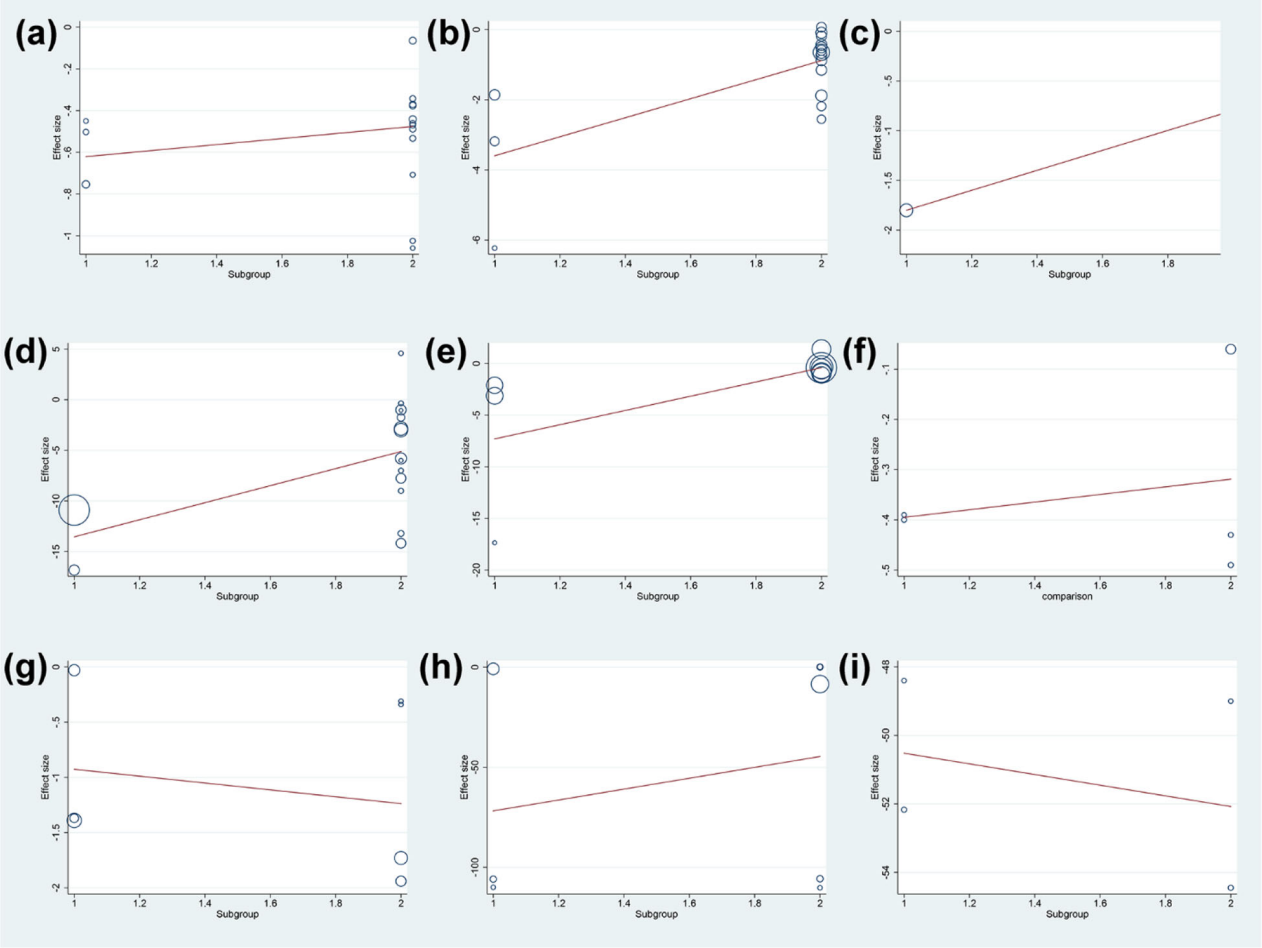
Meta-regression Results of dose **(A)** adverse events of total glucosides of paeony; **(B)** CRP of total glucosides of paeony; **(C)** DAS28 of total glucosides of paeony; **(D)** ESR of total glucosides of paeony; **(E)** RF of total glucosides of paeony; **(F)** CRP of curcumin; **(G)** DAS28 of curcumin; **(H)** ESR of curcumin; **(I)** RF of curcumin.

Meanwhile, the dose was not the source of heterogeneity in DAS28 (P=0.283) and adverse events (P=0.646) of total glucosides of paeony. However, the dose may be the source of heterogeneity in ESR (P=0.021), CRP (P=0.001) and RF (P=0.047) of total glucosides of paeony. In addition, the dose was not the source of heterogeneity in DAS28 (P=0.638), ESR (P=0.553), CRP (P=0.704), RF (P=0.885) (**Figure 16**).

#### 3.19.5 Evidence quality assessment

The evidence quality of total glucosides of paeony and curcumin was assessed by GRADE pro CDT (**Tables 4**, **5**). The recommended rating for all results is low to very low.

**Table 4:** The evidence quality of total glucosides of paeony.

Outcomes	Illustrative comparative risks* (95% CI)		Relative effect (95% CI)	No of Participants (studies)	Quality of the evidence (GRADE)	Comments
	Assumed risk	Corresponding risk				
	Control	Total glucosides of paeony				
**DAS28**		The mean DAS28 in the intervention groups was **0.92 lower** (1.52 to 0.31 lower)		1146 (9 studies)	⊕⊕⊝⊝ **low** ^1^	
**ESR**		The mean ESR in the intervention groups was **6.44 lower** (9.24 to 3.63 lower)		1522 (16 studies)	⊕⊕⊝⊝ **low** ^1,2^	
**CRP**		The mean CRP in the intervention groups was **1.32 standard deviations lower** (1.81 to 0.83 lower)		1513 (17 studies)	⊕⊝⊝⊝ **very low** ^1,2,3^	SMD -1.32 (-1.81 to -0.83)
**RF**		The mean RF in the intervention groups was **2.01 standard deviations lower** (3.01 to 1.01 lower)		984 (10 studies)	⊕⊝⊝⊝ **very low** ^1,2,3^	SMD -2.01 (-3.01 to -1.01)
**Adverse events**	**Study population**		**RR 0.55** (0.44 to 0.69)	1362 (15 studies)	⊕⊕⊝⊝ **low** ^1,2^	
	**242 per 1000**	**133 per 1000** (106 to 167)			
	**Moderate**				
	**250 per 1000**	**138 per 1000** (110 to 172)			

*The basis for the **assumed risk** (e.g. the median control group risk across studies) is provided in footnotes. The **corresponding risk** (and its 95% confidence interval) is based on the assumed risk in the comparison group and the **relative effect** of the intervention (and its 95% CI).

**CI,** Confidence interval; **RR,** Risk ratio;

GRADE Working Group grades of evidence.

**High quality:** Further research is very unlikely to change our confidence in the estimate of effect.

**Moderate quality:** Further research is likely to have an important impact on our confidence in the estimate of effect and may change the estimate.

**Low quality:** Further research is very likely to have an important impact on our confidence in the estimate of effect and is likely to change the estimate.

**Very low quality:** We are very uncertain about the estimate.

^1^ Downgraded one level due to the probably substantial heterogeneity.

^2^ Downgraded one level due to serious risk of bias (random sequence generation, allocation concealment, blinding, incomplete outcomes) and most of the data comes from the RCTs with moderate risk of bias.

^3^ Downgraded one level due to the publication bias.

**Table 5:** The evidence quality of curcumin.

Outcomes	Illustrative comparative risks* (95% CI)		Relative effect (95% CI)	No of Participants (studies)	Quality of the evidence (GRADE)	Comments
	Assumed risk	Corresponding risk				
	Control	Primary outcomes				
**DAS28**		The mean DAS28 in the intervention groups was **1.1 lower** (1.67 to 0.53 lower)		147 (7 studies)	⊕⊝⊝⊝ **very low** ^1,2,3^	
**ESR**		The mean ESR in the intervention groups was **54.67 lower** (88.32 to 21.02 lower)		191 (8 studies)	⊕⊝⊝⊝ **very low** ^1,2,3,4^	
**CRP**		The mean CRP in the intervention groups was **0.35 lower** (0.55 to 0.15 lower)		104 (5 studies)	⊕⊝⊝⊝ **very low** ^1,2,3,4^	
**RF**		The mean RF in the intervention groups was **51.3 lower** (60.59 to 42.01 lower)		60 (4 studies)	⊕⊝⊝⊝ **very low** ^1,2,3,4^	

*The basis for the **assumed risk** (e.g. the median control group risk across studies) is provided in footnotes. The **corresponding risk** (and its 95% confidence interval) is based on the assumed risk in the comparison group and the **relative effect** of the intervention (and its 95% CI).

**CI,** Confidence interval;

GRADE Working Group grades of evidence.

**High quality:** Further research is very unlikely to change our confidence in the estimate of effect.

**Moderate quality:** Further research is likely to have an important impact on our confidence in the estimate of effect and may change the estimate.

**Low quality:** Further research is very likely to have an important impact on our confidence in the estimate of effect and is likely to change the estimate.

**Very low quality:** We are very uncertain about the estimate.

^1^ Downgraded one level due to serious risk of bias (random sequence generation, allocation concealment, blinding, incomplete outcomes) and most of the data comes from the RCTs with moderate risk of bias.

^2^ Downgraded one level due to the probably substantial heterogeneity.

^3^ Downgraded one level due to the total sample size fails to meet the optimal information size.

^4^ Downgraded one level due to the publication bias.

## 4 Discussion

### 4.1 Pomegranate extract for RA

*Punica granatum L.* is rich in vitamins, minerals, organic acids, proteins (79), and also rich in phenolic and flavonoid active ingredients. Pomegranate peel polyphenols mainly include gallic tannin, ellagitannin, ellagic acid, chlorogenic acid, gallic acid, catechin, epicatechin, anthocyanin, ferulic acid and quercetin (80, 81). Studies have shown that pomegranate has been consumed as a medicinal plant for thousands of years and has various properties such as anti-inflammatory, antioxidant, anti-cancer, anti-diabetic, anti-hyperlipidemic, anti-hypertensive and cardiovascular protection (82, 83). In addition, animal (84) and human (85, 86) studies did not report any serious adverse outcomes following pomegranate consumption. Animal studies have shown that ethanolic extract of pomegranate peel can significantly increase paw withdrawal latency and reduce adverse histological changes and arthritis scores (87), and reduce serum RF, MDA, IL-1β and TNFα. Karwasra et al. reported that Pomegranate extract can significantly inhibit hind paw swelling and bone destruction, and reduce complications such as erythema, improve joint inflammation, synovial hyperplasia, inflammatory cell infiltration, periarticular bone resorption, bone erosion and joint space narrowing (88).

*In vitro* studies indicated that pomegranate seed oil and fermented pomegranate juice extract can inhibit COX and lipoxygenase involved in triggering the inflammatory cascade (89). The main fatty acid punicic acid in pomegranate seed oil inhibits prostaglandin biosynthesis (90) and inhibits neutrophil activation and lipid peroxidation outcomes (91). In addition to the inhibitory effect of pomegranate on eicosanoid production, pomegranate also exerts its anti-inflammatory effect by inhibiting the p38-MAPK pathway and the transcription factor NF-κB (89–91). Extracts of phenolic compounds present in pomegranate peel are potential scavengers of diphenylpicrylhydrazine (DPPH) free radicals. This may be because the phenolic substances in the peel extract have strong proton-donating ability and can agglomerate with hydroxyl groups to stabilize free radicals (92, 93). Morvaridzadeh et al. demonstrated that pomegranate juice caused a non-significant increase in TAC and paraoxonase and a non-significant decrease in MDA concentration in humans (94).

The limitation of this systematic review is that it contains only 1 RCT, and they found that compared with placebo, pomegranate extract reduced DAS28 scores, improved joint swelling and tenderness, decreased ESR levels, and increased GPx concentrations.

### 4.2 Quercetin for RA

Quercetin is a flavonoid commonly found in fruits and vegetables such as onions, apples, beans and various berries (95). It has anti-inflammatory, anti-angiogenic, anti-cancer, hepatoprotective, cardiovascular, anti-aging, and neuroprotective potentials (96–103). In a large number of preclinical or clinical studies, quercetin has also been shown to have a certain effect on RA (30, 104). Preclinical studies have found that quercetin can regulate Th17/Treg cell balance, reduce Th17 cell-related cytokines (IL-17A, IL-21 and IL-23), and increase Treg cell-related cytokines (IL-10 and TGF-β), and reduce the level of autoantibodies (105–109). Quercetin also reduces pro-inflammatory cytokines [TNF-α, IL-1β, IL-6, IL-8, prostaglandin (PG) E2, COX-2, inducible nitric oxide synthase (iNOS) and prepro-ET-1] by modulating MAPKs (ERK, p38, JNK), NF-κB and Nrf2/HO-1 signaling pathways, and the lncRNA XIST/miR-485/PSMB8 axis (110–116). In terms of bone protection, quercetin can modulate mTOR, ERK, IκB-α and AMPK signaling pathways and inhibit the expression of receptor activator of NF-κB ligand (RANKL) in FLS (30–32). It can also inhibit the activation of MAPKs (ERK, p38, JNK) and NF-κB signaling pathway to inhibit the expression of MMP-1 and MMP-3 in FLS (117–119).

Only two RCTs reported quercetin in the treatment of RA. Javadi et al., 2017 found that DAS-28 decreased and serum TNF-α levels were significantly reduced after quercetin intervention compared to placebo (28, 29). However, Bae et al., 2009 showed a negative result (30). Because meta-analyses could not be combined, the results need to be interpreted with caution.

### 4.3 Resveratrol for RA

Resveratrol is a natural polyphenolic antioxidant that is abundantly present in a variety of plants, especially red grape skins, and has pharmacological properties, including antioxidants, reactive oxygen species with scavenging, anticancer, and anti-inflammatory effects (120–123). Previous studies have shown that resveratrol has beneficial effects on the occurrence and development of RA (124–126). Resveratrol acts directly on mitochondrial production of ROS. Its ROS scavenging potential may be driven by the dissociative junction of leaked electrons from the respiratory chain (127–129). Several studies have reported that resveratrol can reduce the activity of COX-1, COX-2, and reduce the expression of potent inflammatory parameters such as prostaglandins and leukotrienes (130, 131). In addition, resveratrol reduced TNF-α and IL-1β levels in rats with adjuvant arthritis (132). Cheon et al. demonstrated that resveratrol supplementation significantly reduced inflammation, pannus formation, and cartilage damage in mice with collagen-induced arthritis, and reduced bone destruction (133).

In this systematic review, only 1 RCT reported resveratrol in the treatment of RA. Khojah et al., 2018 found that resveratrol treatment reduced swollen and tender joint counts, decreased DAS28, and decreased serum CRP, ESR, hypocarboxylated osteocalcin, MMP3, TNF-α, and IL-6. More RCTs are needed for further research in the future (31).

### 4.4 Garlic extract for RA

Garlic is a functional food that has been used worldwide for thousands of years. It is rich in bioactive compounds including allicin, ajoene, s-allyl cysteine, s-methyl cysteine, diallyl disulfide (DADS), diallyl sulfide (DAS), alliin, amino acids, polysaccharides and different polyphenols (134–136). The main phenolic compounds are β-resorcinol, gallic acid, pyrogallol, quercetin, rutin and protocatechuic acid (137). Garlic improves immune system function by stimulating certain cell types, such as macrophages and lymphocytes, and reduces cytokine secretion (138). The anti-inflammatory effects of allicin and DAS have been demonstrated in several studies (139, 140). Garlic-derived compounds can reduce serum TNF-α, IL-6 and CRP levels by inhibiting cell signaling pathways, including COX-2 and inhibit NF-κB activation (141, 142). In addition, scientific studies have reported the analgesic and anti-fatigue effects of garlic (143–145). The antioxidant properties of garlic may help reduce pain in patients with rheumatoid arthritis (146). For example, garlic supplements can relieve pain in patients with knee osteoarthritis (OA) (147–150). Hussain and Salalah (149) showed that 900 mg of garlic (capsules) per day for 8 weeks significantly reduced TNF-α concentrations in patients with knee OA (17). Treatment with garlic (400 mg/kg) reduced writhing caused by PG, a dose that showed similar effects to aspirin, one of the most commonly used analgesics (33, 151, 152).

Only one RCT reported Garlic extract in the treatment of RA. Moosavian et al., 2020 found that Garlic extract intervention improved antioxidant levels (increased TAC, decreased MDA), improved quality of life, and improved inflammation (decreased CRP and TNF-α), decreased joint pain and tenderness, and decreased DAS28 compared with the placebo group (32, 33).

### 4.5 Total glucosides of paeony for RA

Total glucosides of paeony are extracted from the dried roots of Paeonia lactiflora (153), including paeoniflorin, hydroxypaeoniflorin, paeoniflorin, paeoniflorin, and benzoylpaeoniflorin (154). Total glucosides of paeony have a wide range of anti-inflammatory and immunomodulatory effects, and have been widely used in the treatment of autoimmune diseases, including RA, systemic lupus erythematosus (SLE), psoriasis, allergic contact dermatitis, etc. (155–159). In terms of intervening in RA, total glucosides of paeony regulate the function and activation of immune cells in RA, reduce the production of inflammatory mediators, and restore abnormal signaling pathways (160, 161). Total Paeoniflorin can balance immune cell subsets (such as macrophage-like synoviocytes, fibroblast-like synoviocytes, etc.). Paeoniflorin can regulate signaling pathways (GPCR pathway, MAPKs/NF-κB pathway, PI3K/Akt/mTOR pathway, JAK2/STAT3 pathway, TGFβ/Smads, etc.) in experimental arthritis FLS (160, 162–165).

This meta-analysis showed that total glucosides of paeony may improve the clinical manifestations of RA (decrease DAS28, ACR20 and ACR70) and inhibit inflammatory (reduce CRP, ESR, RF, IL-6 and TNF-α). Meanwhile, the addition of total glucosides of paeony may reduce the incidence of adverse events.

### 4.6 Tea polyphenols for RA

Tea polyphenols (such as catechins, etc.) are the most abundant in tea (166, 167). Therapeutic benefits of green tea have been seen in neurodegenerative, inflammatory, cardiovascular, and various cancers (168, 169). Catechins have demonstrated their anti-inflammatory effects in many studies related to pathological conditions in which inflammation is a core driver (170). Extensive *in vitro* studies have shown that catechins have promising applications in the treatment of RA, with differential modulation of cartilage, bone, and synovial fibroblast activity (171). In cartilage, catechins have been found to inhibit IL-1β-induced inducible NOS (iNOS) and COX-2 expression by inhibiting IκBα phosphorylation and proteasomal degradation (172, 173). Catechin also inhibits IL-1β-induced phosphorylation of c-Jun, thereby preventing activating protein 1 (AP-1) from binding to DNA (174). Akhtar and Haqqi found that when IL-1β was inhibited, IL-6, IL-8 and TNF-α were also down-regulated due to the inhibition of NF-κB (175). An early study suggests that prophylactic consumption of green tea may help improve inflammation and reduce cartilage destruction associated with different forms of arthritis (176). In bone biology, catechins are thought to reduce the amount of osteoclast formation by reducing osteoblast differentiation (177). Catechin blocks the receptor activator of RANKL-mediated activation of JNK and NF-κB pathways to inhibit the expression of the transcription factor NFATc1 required for osteoclast differentiation (178). Catechins can regulate B cell activating factors belonging to the TNF family (BAFF)/PI3K/AKT/mTOR pathway to induce apoptosis, also in B lymphocytes (179). In regulating apoptosis, catechin treatment selectively downregulated Mcl-1 (anti-apoptotic protein) expression, thereby increasing the sensitivity of synovial fibroblasts to apoptosis (180). Studies have also shown that catechins can reduce the production of MMP-1, MMP-2 and MMP-3 by RA synovial fibroblasts to prevent further cartilage and bone destruction (181, 182). Therefore, although tea and tea polyphenols can neutralize the inflammatory effects of IL-1β and IL-6, they also effectively utilize TNF-α to play its basic function of regulating the uncontrolled proliferation of activated synovial fibroblasts to improve the functional status of arthritis joints.

This meta-analysis showed that tea polyphenols may improve the clinical manifestations of RA (decrease DAS28, ACR20 and ACR70) and inhibit inflammatory (reduce CRP and ESR). Meanwhile, the addition of total glucosides of paeony may not increase the incidence of adverse events.

### 4.7 Puerarin for RA

Puerarin exists in the roots of the genus Pueraria (common name Pueraria), which is isolated from Pueraria and other species (183–185). Numerous health benefits have been attributed to puerarin, namely antioxidant (186), anti-inflammatory (187), neuroprotective (188), liver protection (189), anticancer (190), antidiabetic (191), cardioprotective (192) and anti-atherosclerotic effect (193). In terms of bone protection, puerarin is an effective compound that inhibits bone resorption and improves bone structure. It can stimulate osteoblast differentiation and inhibit osteoclastogenesis at the same time (183, 194). In addition, the Ca 2+ content in the culture supernatant decreased after puerarin treatment. Puerarin can stimulate bone formation and regulate bone metabolism by inhibiting bone resorption (195). Multiple studies have found that puerarin attenuates inflammation and oxidation in mice with collagen antibody-induced arthritis through the TLR4/NF-κB signaling pathway (196). Puerarin derivative (4AC) antioxidant and inhibits TNF-α activity *via* MAPKs/NF-κB signaling pathway in RAW264.7 cells and collagen-induced arthritis rats (197). Puerarin inhibits inflammation and ECM degradation through the Nrf2/HO-1 axis in chondrocytes and reduces pain symptoms in osteoarthritis mice (198).

The anti-atherosclerotic properties of puerarin also include inhibition of lipopolysaccharide or ovalbumin-induced inflammation (199–201), protection of endothelial cells from damage induced by oxidized LDL or Aß40, and reduction of lipid accumulation in vessel walls (199–203). The vasoprotective effect of puerarin inhibits vascular smooth muscle cells (204) and protects against ischemia and reperfusion injury (205–207). This systematic review showed that puerarin may decrease DAS28, ESR, CRP, IL-6.

### 4.8 Hesperidin for RA

Hesperidin is a flavonoid that is abundant in citrus fruits. Hesperidin has many biological functions, including antioxidant, anti-inflammatory, antiviral, and anticancer activities (208). Ahmad et al. found that after treatment with hesperidin, plasma CML and IgG PTD levels were restored to 93% and 16%, respectively, through the free radical scavenging activity of hesperidin, thereby alleviating RA disease by reducing the concentration of AGEs. Therefore, the use of hesperidin may help reduce the severity of RA disease. Umar et al. found that hesperidin may inhibit collagen-induced arthritis by inhibiting free radical load and reducing neutrophil activation and infiltration (209). Qi et al. (210) found that in complete Freund’s adjuvant-induced arthritis in mice, hesperidin inhibited synovial cell inflammation and macrophage polarization by inhibiting the PI3K/AKT pathway. Liu et al. (211) found that hesperidin derivative 11 inhibited the proliferation of fibroblast-like synoviocytes by activating frizzled-related protein 2 secreted in adjuvant arthritis rats. Li et al. found the therapeutic effect of hesperetin on adjuvant arthritis in rats by inhibiting the JAK2-STAT3 signaling pathway (212). Hesperidin also promotes the anti-inflammatory and analgesic activities of Siegesbeckia pubescens makino by inhibiting COX-2 expression and inflammatory cell infiltration (213). This systematic review showed that hesperidin may improve RA symptoms.

### 4.9 Crocus sativus L. extract for RA

Crocus sativus L. extract is well known in herbal medicine and has attracted the attention of researchers for its properties, especially its anti-inflammatory and proliferative abilities in bone and cartilage destructive diseases (214, 215). Among these bioactive components, there are four recognized components that may be associated with the therapeutic potential of saffron, including crocin, saffron flavonoids, saffron aldehyde, and saffron (216, 217). Recent studies have revealed other therapeutic and pharmacological activities of saffron (218–225), such as neuroprotection, neurogenetics, antidepressant, antiapoptotic, antioxidant, and anti-inflammatory. One study found that crocin modulates serum levels of enzymatic and non-enzymatic inflammatory cytokines, including MMP-13, MMP-3, MMP-9, HAases, TNF-α, IL-1β, NF-κB, IL -6, COX-2 and PGE2 and ROS media (226). Crocin also increased levels of GSH, SOD, CAT and GST. In addition, inhibition of the exoglycosidase cathepsin-D and tartrate-resistant acid phosphatase in the bone near the joint by crocin protects bone resorption (226). Rasol et al. found that TNF-α and IL-1β levels were decreased and SOD and GR activities were increased after crocin intervention (226). Hu et al. found that paw swelling and ankle diameter were significantly reduced in crocin-treated rats. Histological analysis also showed reduced inflammation in joints and other organs, such as the spleen. In addition, TNF-α and TGF-β1 levels were decreased in synovial tissue (227). In a similar study, Liu et al. found that MMP-1, MMP-3, and MMP-13 protein expression levels were decreased in RA rats after crocin intervention (228). Li et al. showed similar results, suggesting that crocin had a positive effect on RA-induced rats (229). In an *in vitro* study, Li et al. demonstrated that crocin at 500 µM (5,000 mg/ml) reduced levels of TNF-α, IL-1β, and IL-6 in human FLS. Furthermore, crocin caused lower levels of p-IκBα, p-IκB kinase α/β and p65 expression, demonstrating its effect on the NF-κB pathway (230). Wang et al. showed that crocin inhibits Wnt/β-catenin and Wnt signaling pathways to reduce pain-related cytokines, and glial activation may alleviate RA-induced neuropathic pain in rats (231).

However, this meta-analysis showed that the efficacy of Crocus sativus L. extract may not be significantly different from the control group.

### 4.10 Ginger extract for RA

Ginger has been cultivated in China and other countries around the world since ancient times as a source of medicinal plants for spice and therapeutic benefits (232). The main components of ginger are 6-shogaol, ginger oil terpene, water fennel, camphor terpene, gingerol, eucalyptus, etc. (232–234). Evidence suggests that consuming ginger may help relieve joint pain associated with RA (235). Kiuchi et al. discovered the potential of ginger to inhibit the synthesis of prostaglandins, which are key to inflammation. Further research found that ginger exhibits anti-inflammatory activity by inhibiting the biosynthesis of prostaglandins and leukotrienes (236). Ribel-Madsen et al. (237) conducted the *in vitro* anti-inflammatory effect of ginger, and ginger-treated synoviocytes showed similar inhibitory effects as betamethasone by inhibiting the production of cytokines IL-1 and IL-6. Yang et al. observed the analgesic and anti-inflammatory effects of 6-gingerol (238). Ojewole observed potential analgesic and anti-inflammatory activity of ginger, which can be used to reduce pain and inflammation caused by arthritis (239). Srivastava et al. observed the antiarthritic activity of ginger in patients who independently experienced RA, OA and muscle discomfort (240). The beneficial effect of ginger on reducing RA-related pain may be due to the inhibition of prostaglandin and leukotriene biosynthesis (240). van Breemen et al. found that 10-gingerol, 8-shogaol, and 10-shogaol strongly inhibited COX 2, thereby significantly reducing inflammation (241). In addition, ginger can inhibit the biosynthesis of leukotrienes by inhibiting 5-lipoxygenase (242). Nurtjahja-Tjendraputra et al. also demonstrated the inhibitory effect of ginger on COX-1 activity (243). One study found that components of ginger significantly inhibited the release of pro-inflammatory cytokines (IL-12, TNF-α and IL-1 β) and pro-inflammatory chemokines in LPS-induced macrophages (244). 6-Gingerol significantly inhibits Ikβα phosphorylation, NF-κβ nuclear activation and PKC-α translocation, which in turn inhibits Ca mobilization and disruption of mitochondrial membrane potential in LPS-stimulated macrophages, thereby inhibiting inducible nitric oxide synthase and TNF-α express and reduce inflammation (245). This systematic review showed that Ginger extract may decrease DAS28.

### 4.11 Cinnamon extract for RA

Cinnamon, one of the most commonly used spices in the world and one of the oldest herbal medicines used to treat certain diseases and inflammations, has been used to treat RA in China for nearly 2000 years (246). Cinnamon can modulate immune system function by modulating anti-inflammatory and pro-inflammatory gene expression (247–249). Cinnamaldehyde is the main active component of cinnamon, and its anti-inflammatory effect has been observed in several studies (250–252). Several other flavonoids, including anti-inflammatory hesperidin and quercetin, have also been extracted from cinnamon (253, 254). Scientific evidence suggests that cinnamon extract can be used to modulate the immune system, as well as prevent and treat inflammation (255). The beneficial effects of cinnamon extract and its polyphenols on reducing serum levels of TNF-α, CRP and IL-6, as well as improving clinical symptoms and antioxidant activity have been reported in animal models (256). *In vitro* experiments also demonstrated the beneficial effects of cinnamon polyphenols in improving immune responses by modulating the expression of pro- and anti-inflammatory cytokine genes (257, 258). In experimental arthritis studies, cinnamaldehyde in cinnamon significantly inhibits joint disease in experimentally arthritic animals. Cinnamaldehyde can not only significantly reduce the IL-6 content of inflammatory mediator TNF-α in peripheral mononuclear cells of RA patients (259), but also inhibit the release of IL-1β and matrix MMP-13 from synovial fibroblasts in arthritis patients (260). Cinnamaldehyde can significantly reduce the levels of TNF-α, IL-6 and IL-1β in the peripheral blood of collagen-induced arthritic rats, and significantly increase the content of the anti-inflammatory factor IL-10, exerting a systemic anti-inflammatory effect (261). Liu et al. found that cinnamaldehyde may affect the production of IL-1β by inhibiting HIF-1α, and may affect the maturation of IL-1β by regulating the formation of NLRP3 inflammasome through the succinate/HIF-1α axis (262).

This systematic review showed that Ginger extract may decrease DAS28, the number of tender and swollen joints, and serum CRP and TNF-α levels.

### 4.12 Sesamin for RA

Sesame is an important traditional health food that has been used in Asian countries for thousands of years to improve nutritional status and prevent various diseases (263). Sesame seeds contain high amounts of oil and protein, as well as various lignans (such as sesamin) (264, 265). Several studies have shown that sesamin (one of the most abundant lignans in sesame) has various physiological functions, including antioxidant, antihypertensive, anti-obesity and hypolipidemic effects (266–269). The anti-inflammatory properties of sesame compounds have been reported in rat models (270). The results of another study showed that sesamin inhibited lipopolysaccharide-induced inflammation by inhibiting p38 mitogen-activated protein kinase and NF-kB, which are the major pathways regulating cytokine production. Based on this result, sesamin may also prevent cartilage degeneration in other joint diseases such as RA (271, 272).

This systematic review showed that sesamin may improve the joint pain, decrease the number of tender joints, and decrease serum hs-CRP, TNF-α and COX-2 levels.

### 4.13 Cranberry extract for RA

Cranberry (Vaccinium macrocarpon) juice has strong antioxidant activity, mainly containing polyphenolic compounds such as flavonols (myricetin and quercetin), anthocyanins and procyanidins (273). Clinical studies have shown that cranberry juice has beneficial effects on biomarkers of cardiovascular disease risk (274, 275). Several intervention studies have found that cranberry has beneficial effects on biomarkers of oxidative stress, dyslipidemia, and inflammation in healthy people (276, 277) and in patients with type 2 diabetes (278) and metabolic syndrome (279). Studies have found that quercetin (flavonol), which is abundant in cranberry, can lead to a significant down-regulation of the nuclear factor kappa B (NF-κB) pathway (280). Additionally, resveratrol has been shown to be another abundant polyphenol in cranberries. It can inhibit the expression of inflammatory genes related to cardiovascular disease by activating NF-κB and Janus kinase/signal transducer and transcriptional pathway activator in cultured cells (273). NF-κB regulates the expression of many pro-inflammatory genes, including adhesion molecules, IL-6, and TNF-α. Other components present in cranberries, such as proanthocyanidins, anthocyanins, hydroxycinnamic acid, and acetylsalicylic acid, inhibit NF-κB activation (275).

This systematic review showed that Cranberry extract may decrease DAS28, and serum ESR and CRP.

### 4.14 Olive extracts and oil for RA

The current study found that olive oil contains high amounts of polyphenolic compounds. Numerous studies have found polyphenolic extracts of olive oil as antioxidants to prevent and treat cardiovascular disease and prevent certain types of cancer, as well as reduce the incidence of coronary heart disease and stroke (281–283). Furthermore, it has been shown to inhibit IL-1β-induced MMP, TNF-α and IL-6 production in the SW982 human synovial fibroblast cell line (284). Polyphenol extracts also down-regulated COX-2 and mPGE-1 induced by IL-1β. In addition, it inhibits MAPK phosphorylation and NF-κB activation (285, 286). This systematic review showed that Olive oil may decrease DAS28, relieve joint pain and decrease the number of painful joints and the number of swollen joints. The study also found that a Mediterranean diet centered on olive oil can reduce the risk of autoimmune diseases such as RA (287, 288), which is strong evidence in the treatment of RA.

### 4.15 Curcumin for RA

Curcumin, a yellow pigment and active component of turmeric (Curcuma longa), is one of the well-known natural compounds with a wide range of pharmacological activities and potential immunomodulatory properties (289, 290). Curcumin effectively inhibits the production of several pro-inflammatory mediators by mediating multiple inflammatory signaling pathways (such as JAK-STAT signaling pathway/P38 MAPK signaling pathway, NF-κB signaling pathway, etc.), including TNF-α, IL-1β, IL-6, IL-12 and IL-8 (291–294). Curcumin has a high ability to modulate inflammation in RA by suppressing pro-inflammatory immune cell populations and suppressing the production of immune-inflammatory cytokines and chemokines (295, 296). Studies have found that curcumin can significantly block the expression of IL-6 in FLS stimulated by IL-1β in RA patients (297). Curcumin can also reduce the protein expression levels of IL-6, IL-8, MCP-1, MMP-1 and MMP-3 in FLS of RA patients (298). Several studies on animal models of RA also confirmed this finding. Curcumin can reduce the production of pro-inflammatory cytokines, including TNF-α, IL-1β, IL-6 and MCP-1 (299–304). Curcumin has also been found to inhibit the activation, proliferation and differentiation of naive CD4+ T cells into T helper (TH) 1 and TH 17 subtypes. These two cells play a key role in the pathogenesis of RA by producing key pro-inflammatory cytokines including TNF-α, IFN-γ, IL-17, IL-21 and IL-23 and are responsible for joint and bone destruction (305, 306). In addition, curcumin has a strong ability to induce regulatory T cell (Treg) differentiation and inhibit TH1- and TH17-mediated inflammatory responses (306). This meta-analysis showed that curcumin may decrease DAS28, CRP, ESR and RF.

### 4.16 Mechanisms of other dietary polyphenols with medicinal potential

Dietary polyphenols are currently widely used as alternative therapies, and preclinical and clinical studies have demonstrated their efficacy in the treatment of RA. In addition to the 15 dietary polyphenols (Cinnamon extract, Cranberry extract, Crocus sativus L. extract, Curcumin, Garlic extract, Ginger extract, Hesperidin, Olive oil, Pomegranate extract, Puerarin, Quercetin, Resveratrol, Sesamin, Tea polyphenols, Total glucosides of paeony) in this study, there are many other polyphenols in the world. The reason why this study did not include other polyphenols is that this study is about the systematic review and meta-analysis of RCTs, so these animal experiments were not included. In order to further provide future researchers with reference information for dietary polyphenols in the treatment of RA, we summarized dietary polyphenols with potential medicinal value. Dietary polyphenols derived from dietary fruits, vegetables and natural herbs for anti-rheumatic activity in the treatment of RA include Stilbenes, Phenolic acids, Flavonoids, etc. (307–310). Among them, phenolic acids such as benzoic acid and cinnamic acid (311). Hydroxybenzoic acids (including gallotannins and ellagitannins) also exhibit anti-RA effects (312). Natural flavonoids also exhibit strong anti-RA activity, such as anthocyanins, flavanols (catechins, epigallocatechin gallate), flavonoids (luteolin, apigenin), flavanones (naringenin), flavonols (quercetin and kaempferol), isoflavones (genistein, daidzein and glycitein) (313, 314). Some herbs have also been shown to exhibit prominent anti-RA activity, including *Adhatoda vasica* Nees (pyrroloquinazoline), *Ajuga bracteosa* wall (Withaferin-A, Ajugarin-I), *Aconitum carmechaeli* Debeaux (aconitine, benzoylmethasone), *Barleria prionitis* (triterpenoids), pine (pinitol) (315). These polyphenols play an anti-RA role mainly by regulating immune-inflammation-related signaling pathways, anti-oxidation-related signaling pathways and triggering synoviocyte/pathogenic immune cell programmed death (apoptosis, pyroptosis, ferroptosis) (316–319). At present, the most studied pathways are immune inflammation-related signaling pathways to reduce bone and joint damage, such as NFKB signaling pathways, TLR signaling pathways, TNF signaling pathways, etc. (320, 321). The antioxidant-related signaling pathways regulated by these polyphenols mainly include SIRT1 signaling pathway, Keap1-Nrf2-ARE pathway, NOX4/ROS signaling pathway, HO-1 signaling pathway and iNOS signaling pathway (320, 322, 323). These dietary polyphenols can also regulate programmed cell death signaling pathways to treat RA, such as MAPK pathway or PI3K/Akt signaling pathway and epigenetics-related regulatory signaling pathways (316, 324, 325).

### 4.17 Strength and limitations

The strengths of this research are that compared with previous studies, this study included the results of a meta-analysis and included more RCTs (48 RCTs were included in this study and involved 15 dietary polyphenols: Cinnamon extract, Cranberry extract, Crocus sativus L. extract, Curcumin, Garlic extract, Ginger extract, Hesperidin, Olive oil, Pomegranate extract, Puerarin, Quercetin, Resveratrol, Sesamin, Tea polyphenols, Total glucosides of paeony). Sensitivity analysis, publication bias assessment, meta-regression, and quality of evidence ratings were also performed in this meta-analysis.

The limitations of this research are that: (1) There is obvious heterogeneity in outcomes. According to meta-regression, part of the heterogeneity of total glucosides of paeony and curcumin may come from drug dosage and intervention time. The remaining heterogeneity may be due to the selection of the population, the choice of dietary polyphenol preparations for treatment of dietary polyphenols, and information biases during data collection. (2) Although a total of 48 RCTs were included, except for total glucosides of paeony, each type of dietary polyphenol supplements included no more than 10 RCTs, and the number of participants in each RCT was mostly less than 100. (3) The RCT languages included in this study were only Chinese and English, and no RCTs in other languages were found, which may have an impact on the results. (4) The follow-up time of the RCTs ranged from 8 to 48 weeks, and there was no observation for more than 3 years and before 8 weeks, which may affect the generality of the results. (5) The RCTs in this research have a high risk of bias. The authors of some RCTs are funded or employed by drug manufacturers, which may introduce bias.

### 4.18 Inspiration for the future

Through the systematic review and meta-analysis of this study, it can be found that the research on the efficacy and mechanism of various dietary polyphenols in relieving RA has made significant progress. Among them, curcumin and total glucosides of paeony have improved the treatment of rheumatoid arthritis. Symptoms, inhibition of inflammation and other outcome indicators. In general, dietary polyphenols can be used as an effective anti-inflammatory drug in the adjuvant treatment of RA, and can be used for daily dietary supplementation with relatively good safety.

However, there are still some knowledge gaps in current clinical research and basic research, which need to be addressed in future research. In terms of basic research, most previous studies have cultured a single phenolic compound or a mixture of multiple phenolic compounds with synovial cells, lacking pharmacokinetic studies targeting dietary polyphenols in animal studies. This situation is even more lacking in clinical studies, and few clinical studies have explored the pharmacokinetics of dietary polyphenols in RA patients (such as, absorption and metabolism of polyphenols, and interactions with gut microbiota). For example, in previous studies, most studies using animal or cell culture models focused on the biological activity of polyphenols themselves. Moreover, few studies have focused on the microbial metabolism of polyphenols. In particular, the current study (326–328) showed that the α-diversity and β-diversity of gut microbiota in RA patients were significantly lower than those in healthy people. Among them, Bacteroidetes and Shiga toxoid-producing Escherichia coli were more abundant in RA patients, in contrast, Lactobacillus, Ravivibacterium isoprawizia, Enterobacter and Stinkbacter were less abundant in the RA group. More studies have found that the intestinal flora can affect the development of RA by regulating immune molecules (including immune cells (such as regulatory B cells and CD4 T helper T cells, etc.), immune factors, etc.) (329–332). Therefore, research on the interaction between dietary polyphenols and gut microbiota of RA disease is the main direction of future basic and clinical research. In addition, most dietary polyphenols are degraded by gut flora in the colon due to poor polyphenol absorption. The biological activity of dietary polyphenols may be partially attributed to microbial metabolites. The exact role or biological activity of microbial metabolites should be focused on in future studies.

### 4.19 Prospects

Dietary polyphenols are a group of biologically active phytochemicals with a wide range of sources, diverse structures, low toxicity, and good biological activity. In terms of curative effect in the treatment of RA, it is mainly attributed to the immuno-inflammation modulating activity of dietary polyphenols, which provides a new option for the comprehensive management strategy of RA, and dietary polyphenols also alleviate the adverse reactions of DMARDs. Since these dietary polyphenols are ubiquitously present in the diet, medication adherence may be better in RA patients. Meanwhile, studies have found that some dietary polyphenols can reduce the side effects of glucocorticoids (such as bone destruction, drug resistance, etc.) (333–338). Therefore, the current preliminary preclinical data on dietary polyphenols indicate that polyphenols have great potential in the treatment of RA, and the dietary types that have been carried out RCTs are mainly Cinnamon extract, Cranberry extract, Crocus sativus L. extract, Curcumin, Garlic extract, Ginger extract, Hesperidin, Olive oil, Pomegranate extract, Puerarin, Quercetin, Resveratrol, Sesamin, Tea polyphenols, Total glucosides of paeony. In addition, there are many dietary polyphenols such as rutin, chlorogenic acid, anthocyanins, luteolin, lignans, etc., which need extensive clinical research in the future to provide more clinical evidence for the future clinical treatment of RA. Dietary polyphenols have multi-target effects on signaling pathways in RA, so it is important to further explore the pharmacological mechanism of these dietary polyphenols, which will also provide the molecular structure core of lead compounds or new drugs for RA drug development. Based on this systematic review and meta-analysis, we look forward to clinicians and patients using dietary polyphenols as adjunctive therapy or in combination with other current DMARDs in a comprehensive management strategy for RA. In addition, since some polyphenols are mainly derived from herbs, it is necessary in clinical practice to study the pharmacokinetic results of the interaction of herbs as a multi-component drug (not only polyphenols) with DMARDs to prevent potential adverse drug reactions. Some polyphenol active substances have been formulated into new delivery systems to improve solubility, permeability and enhance the therapeutic effect in preclinical studies due to problems such as absorption. In the future, it is necessary to explore the efficacy and safety of this new type of administration.

## 5 Conclusion

Although the number of RCTs on dietary polyphenols is limited, existing evidence shows their potential benefits, mainly increasing DAS28, reducing CRP and ESR, and improving oxidative stress, etc. However, given the small number of patients recruited, the study designs varied widely between RCTs and the characteristics of RA patients varied; it is difficult to immediately extrapolate these results to RA patients in general. More RCTs are needed in the future to determine the efficacy and safety of dietary polyphenols.

## Data availability statement

The original contributions presented in the study are included in the article/**Supplementary Material**. Further inquiries can be directed to the corresponding author.

## Author contributions

ZL, WaX, QH and WeX are responsible for the study concept and design. ZL, WX, QH, WeX, HW, HL, HG, YC, MY, XY, LZ, KY, YD, ZH are responsible for the data collection, data analysis and interpretation; ZL, WaX and KY drafted the paper; ZH and WeX supervised the study; all authors participated in the analysis and interpretation of data and approved the final paper.
