# Supplementary material for: Efficacy and safety of dietary polyphenols in rheumatoid arthritis: A systematic review and meta-analysis of 47 randomized controlled trials
Source: Front Immunol. 2023 Mar 22;14:1024120. doi: 10.3389/fimmu.2023.1024120 (PMC10073448; doi:10.3389/fimmu.2023.1024120)
Supplement: Supplementary file 17 [file Table_1.docx]

**Table S1.** Search Strategies for Pubmed and Embase

| **PubMed** | ((Polyphenols OR Polyphenol OR Provinols) OR (Curcumin OR Curcumas OR Tumeric OR Tumerics OR Turmeric OR Turmerics OR Curcuma zedoaria OR Curcuma zedoarias OR zedoaria, Curcuma OR Zedoary zedoaria OR Zedoary zedoarias OR zedoaria, Zedoary OR Curcuma longa OR Curcuma longas OR longa, Curcuma OR Curcuma Longa) OR (resveratrol OR resveratrols OR 3,4',5-stilbenetriol OR 3,5,4'-trihydroxystilbene OR trans-resveratrol-3-O-sulfate OR SRT 501 OR SRT501 OR SRT-501 OR trans-resveratrol OR cis-resveratrol OR resveratrol-3-sulfate) OR (Naringenin OR naringenin-7-sulfate) OR (Anthocyanin OR Anthocyanins OR Leucoanthocyanidins OR Anthocyanidin) OR (Hesperidin OR Hesperetin 7-Rhamnoglucoside OR 7-Rhamnoglucoside, Hesperetin OR Hesperetin 7 Rhamnoglucoside OR Hesperetin-7-Rutinoside OR Hesperetin 7 Rutinoside OR Hesperidin 2S) OR (Catechin OR Cianidanol OR Catechinic Acid OR Catechuic Acid OR Catergen OR Zyma OR Epicatechin OR KB-53 OR KB 53 OR KB53 OR Z 7300 OR Cyanidanol-3 OR Cyanidanol 3) OR (Silymarin OR Silimarin OR Karsil OR Legalon OR Carsil) OR Cinnamon extract OR Cranberry extract OR Crocus sativus L. extract OR Garlic extract OR Ginger extract OR Hesperidin OR Olive oil OR Pomegranate extract OR Puerarin OR Sesamin OR Tea polyphenols OR Total glucosides of paeony OR dietary polyphenols))  AND  (Rheumatoid arthritis OR Arthritis, Rheumatoid)  AND  (random* controlled trial [pt] OR controlled clinical trial* [pt] OR randomized [tiab] OR placebo [tiab] OR drug therapy [sh] OR random* [tiab] OR trial* [tiab] OR group* [tiab])  NOT  (animals [mh] NOT humans [mh]) |
| --- | --- |
| **EMBASE** | 1 Polyphenols/  2 Polyphenol/  3 Provinols/  4 1-3/or  5 Curcumin/  6 Curcumas/  7 Tumeric/  8 Tumerics/  9 Turmeric/  10 Turmerics/  11 Zedoary zedoaria/  12 Zedoary zedoarias Curcuma longa/  13 Curcuma zedoaria/  14 Curcuma zedoarias/  15 Curcuma longas/  16 Curcuma Longa/  17 5-16/or  18 resveratrol/  19 resveratrols/  20 trans-resveratrol-3-O-sulfate/  21 trans-resveratrol/  22 cis-resveratrol/  23 resveratrol-3-sulfate/  24 18-23/or  25 Naringenin/  26 naringenin-7-sulfate/  27 25 or 26  28 Anthocyanin/  29 Anthocyanins/  30 Leucoanthocyanidins/  31 Anthocyanidin/  32 28-31/or  33 Hesperidin/  34 Hesperetin 7-Rhamnoglucoside/  35 7-Rhamnoglucoside, Hesperetin/  36 Hesperetin 7 Rhamnoglucoside/  37 Hesperetin-7-Rutinoside/  38 Hesperetin 7 Rutinoside/  39 Hesperidin 2S/  40 33-39/or  41 Catechin/  42 Cianidanol/  43 Catechinic Acid/  44 Catechuic Acid/  45 Catergen/  46 Zyma/  47 Epicatechin/  48 KB-53/  49 KB 53/  50 KB53/  51 Z 7300/  52 Cyanidanol-3/  53 Cyanidanol 3/  54 41-53/or  55 Silymarin/  56 Silimarin/  57 Karsil/  58 Legalon/  59 Carsil/  60 55-59/or  61 4 or 17 or 24 or 27 or 32 or 40 or 54 or 60  62 exp arthritis, rheumatoid/  63 (felty$ adj2 syndrome).tw.  64 (caplan$ adj2 syndrome).tw.  65 rheumatoid nodule.tw.  66 (sjogren$ adj2 syndrome).tw.  67 (sicca adj2 syndrome).tw.  68 still$ disease.tw.  69 bechterew$ disease.tw.  70 (arthritis adj2 rheumat$).tw.  71 62-70/or  72 random$.ti,ab.  73 factorial$.ti,ab.  74 (crossover$ or cross over$ or cross-over$).ti,ab.  75 placebo$.ti,ab.  76 (doubl$ adj blind$).ti,ab.  77 (singl$ adj blind$).ti,ab.  78 assign$.ti,ab.  79 allocat$.ti,ab.  80 volunteer$.ti,ab.  81 crossover procedure.sh.  82 double blind procedure.sh.  83 randomized controlled trial.sh.  84 single blind procedure.sh.  85 72-84/or  86 61 and 71 and 85 |
